# Polymorphic regenerated silk fibers assembled through bioinspired spinning

**DOI:** 10.1038/s41467-017-00613-5

**Published:** 2017-11-09

**Authors:** Shengjie Ling, Zhao Qin, Chunmei Li, Wenwen Huang, David L. Kaplan, Markus J. Buehler

**Affiliations:** 1 Department of Civil and Environmental Engineering, Massachusetts Institute of Technology, Cambridge, MA 02139 USA; 2 Department of Biomedical Engineering, Tufts University, Medford, MA 02155 USA

## Abstract

A variety of artificial spinning methods have been applied to produce regenerated silk fibers; however, how to spin regenerated silk fibers that retain the advantages of natural silks in terms of structural hierarchy and mechanical properties remains challenging. Here, we show a bioinspired approach to spin regenerated silk fibers. First, we develop a nematic silk microfibril solution, highly viscous and stable, by partially dissolving silk fibers into microfibrils. This solution maintains the hierarchical structures in natural silks and serves as spinning dope. It is then spun into regenerated silk fibers by direct extrusion in the air, offering a useful route to generate polymorphic and hierarchical regenerated silk fibers with physical properties beyond natural fiber construction. The materials maintain the structural hierarchy and mechanical properties of natural silks, including a modulus of 11 ± 4 GPa, even higher than natural spider silk. It can further be functionalized with a conductive silk/carbon nanotube coating, responsive to changes in humidity and temperature.

## Abstract (web summary)

Natural silk fibers are produced using a simple and green approach compared to alternative synthetic methods. Here, the authors show a bioinspired approach to spin regenerated silk fibers using anisotropic liquid crystals and dry spinning, resulting in remarkably robust fibers.

## Introduction

Animal-produced silks, produced by spiders and silkworms, have attracted the intense attention of scientists and engineers for more than a century, not only because of their marvelous mechanical properties, but also due to their diverse applications in textiles, optics, environmental engineering, and biomedicine^1–4^. In addition to in-depth studies of the physical properties and functions of natural silk fibers, experimental attempts have been pursued to mimic the natural process of producing robust regenerated silk fibers (RSFs) to emulate the properties of natural silk fibers^5–7^. Wet spinning techniques, ejection of the spinning dope into a coagulation bath (often containing alcohols or salts), are the most common approach to generate RSFs^6–8^. However, these methods are complicated, generally include dissolution, dialysis, concentration, spinning and post-treatment processes, and most of the steps are time-consuming, energy-intensive, and require relatively large quantities of solvent.

In contrast, natural spinning is an anisotropic (liquid crystal)-based dry-spinning process^5, 9–11^. Spiders and silkworms construct webs and cocoons by directly spinning a pre-assembled nematic silk protein dope, which is solidified immediately to a fiber once it leaves the spinneret^5, 10, 11^. All of these processes are conducted under physiological and ambient conditions without any additional immobilization and post-processing steps^5, 9–11^. *Bombyx mori* (*B. mori*) silkworm spinning process as an example. The main structure of silk fibroin is synthesized at the epithelial wall of posterior silk gland (the tail of gland) with a concentration around 12 wt%^12^. Next, the fibroin moves to the wider middle division (sac or ampulla) with an increase in concentration (~25 wt%^12^) and assembles to a micelle-like configuration with anisotropic liquid-crystalline properties^5, 10^. The liquid crystallinity allows the molecules to flow in a pre-aligned manner and to further align along the flow axis during the passage through the spinning duct. Finally, silk fiber formation occurs under shear stress and dehydration conditions during the pulling out of the fiber from the spigot^5, 9–11^.

Several reported dry-spinning technologies^13–21^, spinning processes by which solidification of the fiber occurs due to evaporation of a volatile solvent^7^, have shown advantages for mimicking this fantastic natural spinning process, including ease of operation and relatively low cost. However, as-spun RSFs produced by these methods are brittle and have poor mechanical properties. Therefore, they still require complex post-processing treatments (e.g., dehydration and crystallization processes^7^) to generate useful fibers. This drawback deeply hinders the application of these methods, and, more importantly, all of these attempts (including wet and dry spinning) only focus on reproducing the mechanical properties of natural silks, and pay less focus on retaining the hierarchical structures of silks, a key feature in the properties of the natural protein fibers^22–27^.

On the basis of the anisotropic dry-spinning features of natural spinning, here we elaborate a facile bioinspired spinning strategy to collect RSFs in ambient environmental conditions. The RSFs are formed directly after extruding or pulling silk microfibril (SMF) solution from a spinneret and no post-processing is required. The resultant as-spun RSFs retain the hierarchical architecture and physical properties of natural silks, exhibiting excellent mechanical properties. In addition, this bioinspired spinning approach can be applied to generate polymorphic hierarchical RSFs, such as spiral and helical fibers, and even to build refined 2D and 3D architectures. Finally, we show how the scope of these RSFs can be amplified by adding conductive silk/carbon nanotube coatings, which are suitable for generating humidity and temperature sensors with potential in wearable device/biosensor applications due to the robust silk fibers as a foundation.

## Results

### Bioinspired spinning strategy

**Fig. 1:**
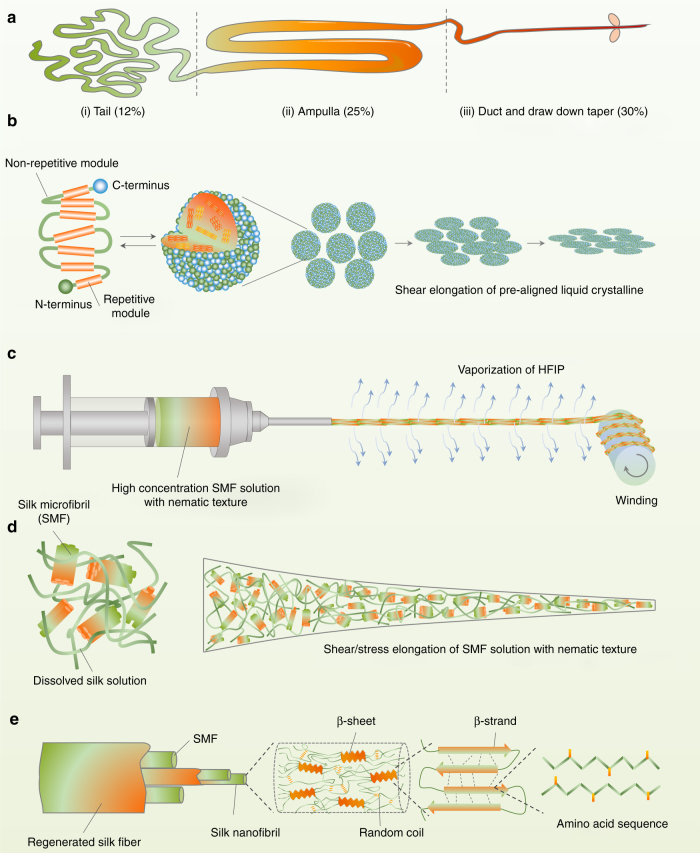
The natural and bioinspired spinning process. **a** Illustration of a silkworm spinning gland divided into three parts according to the evolution of silk protein during spinning. **b** Schematic model of the natural silk fiber assembly mechanism occurring along the spinning apparatus. The scheme is adapted from ref. ^28^, with permission from Elsevier. The silk proteins are synthesized in the tail and are transferred to ampulla with increased concentration. In this region, the silk proteins are assembled to micelle-like configurations with anisotropic liquid-crystalline properties. Finally, silk fiber formation occurs under shear stress and dehydration conditions during pulling out the nematic silk proteins from the spigot. **c** Illustration of the bioinspired spinning process. The nematic silk microfibril solution can be directly assembled into RSFs without additional treatment. **d** Schematic of the SMF evolutionary process during spinning. The SMFs are aligned in the spinning jet (or fiber) axis direction under the shear/stress elongation. **e** Schematic of the hierarchical structure of RSFs. There are at least five structural hierarchy levels in RSF

Same as the natural spinning of *B. mori* silkworm (Fig. 1a, b), a critical factor in bioinspired spinning (Fig. 1c–e) is to prefabricate a spinning dope with suitable rheological properties, which has high viscosity along with stability^29^. Previous attempts focused on increasing the concentration of silk in solution, with different single-solvent, binary-solvent systems, such as HFIP^30, 31^, HFA^32, 33^, NMMO/H_2_O^34–38^, LiBr/H_2_O^39–42^, and CaCl_2_/formic acid^15^ assessed (details can be found Supplementary Table 1). However, the structural hierarchy of natural silks, an important element in determining bulk material properties^22–27^, is destroyed during these dissolution processes^43^. Recently, we found that HFIP can partially dissolve *B. mori* silkworm cocoon silk fibers to microfibrils with diameters of 5–50 µm and contour lengths of 50–500 µm after incubating silk fiber/HFIP (weight ratio, 1:30) mixtures at 60 °C^44, 45^.

**Fig. 2:**
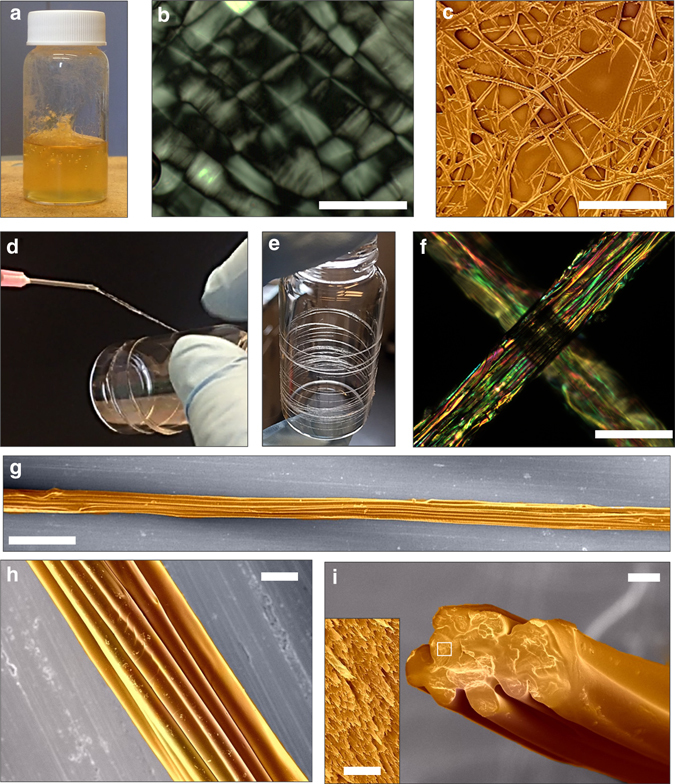
Visual appearance and structural characterization of the regenerated silk spinning dope and resultant RSFs. **a**–**c** Visual appearance (**a**), polarized light microscopy image (**b**), and SEM image (**c**) of *B. mori* silk fiber /HFIP mixture with a weight ratio of 1:20 after incubation at 60 °C for 15 days. After 15 days the silk fiber partial dissolved to microfibrils with diameters of 5–10 µm and contour lengths of several hundreds to thousands of micrometers. The resultant SMF/HFIP mixture was a uniform, highly viscous solution with nematic liquid-crystal-like texture. **d** The photograph to show the facile bioinspired spinning process. The nematic SMF/HFIP solution can be directly reeled to form RSFs. **e**, **f** Visual appearance (**e**) and polarized light microscopy image (**f**) of as-spun RSFs. **g**–**i** SEM images of as-spun RSFs. The images **h** and **i** are a top view and cross-sectional SEM images of RSF, respectively. The RSF is constituted by highly oriented and bound SMFs. The *inset* of the image **i** is high-resolution SEM image of a cross-section of RSF. Well-organized silk nanofibrils are observed. False color is used in SEM images. *Scale bars*, 50 µm (**b**), 200 µm (**c**), 100 µm (**f**), 200 µm (**g**), 20 µm (**h**), 20 µm (**i**), and 2 µm (*inset* of **i**)

Herein, we use the same dissolution system but increase the weight ratio of silk fiber/HFIP to 1:20 and extend the incubation time to 7–15 days (Fig. 2a–c and Supplementary Fig. 1). These new conditions enhance the concentration and viscosity of the SMF solution; more suitable for generating a spinning dope. During the incubation, the HFIP gradually permeated into the silk fibers from the defects and ends, and partially dissolved the sheath layer into silk fibroin polymer chains^44^. After 4 days, the silk fiber/HFIP mixture formed a pulp blend (Supplementary Fig. 1b). The silk fibers were dissolved and cut into shorter fibers with centimeter length and 5–20 µm in diameter. After 15 days, the silk fibers are partially dissolved to form the microfibrils, but in this case the SMFs present in smaller diameters (5–10 µm) and longer contour lengths (several hundreds to thousands of micrometers) (Fig. 2c). Importantly, the resultant silk fiber/HFIP mixture presents as a uniform viscous solution (Fig. 2a) with nematic liquid-crystal-like texture (Fig. 2b, Supplementary Fig. 2). Specifically, analogous to the characteristic of nematic silk proteins in silk glands^5, 11^, these SMFs form a substance that flows as a liquid but maintains some of the orientational order characteristics of a crystal (Fig. 1d). These liquid crystals allow the viscous SMFs to flow through the spinneret to form complex alignment patterns under mild shear and stress. The result is an SMF solution that can be easily transformed into a hardened fiber with moderate external forces and relatively simple devices. For instance, we can directly collect the highly oriented uniform fibers by continuous extrusion with a flow rate of 20 ml h^−1^ or forcibly reeling the SMF solution with reeling speeds of 4–14 mm s^−1^ (Fig. 2d–g). The longest continuously spun RSF reached up to tens of meters under the reeling speed of 4 mm s^−1^, despite a few defects found on the surface. Figure 2h and i present a typical surface and cross-section morphologies of the RSFs with tightly stacked SMFs. The SMFs fuse together and align along the fiber axis without gaps or cracks among the SMFs in a cross-section direction. Fourier transform infrared spectroscopy (FTIR) characterization reveals that the RSFs are mainly composed of β-sheet (crystalline) structures. The deconvolution of the amide I band provides an estimation of β-sheet structure in the RSFs of 34 ± 5% to 45 ± 3%, while that of the degummed *B. mori* silkworm cocoon silk fibers is 38 ± 4% (Supplementary Fig. 3 and Supplementary Table 2).

### Mechanical performance of RSFs

**Fig. 3:**
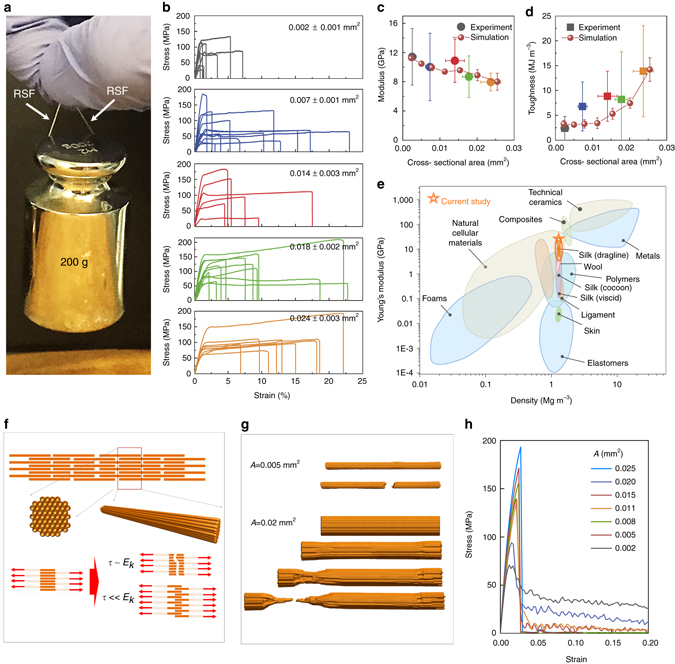
Mechanical properties of RSFs. **a** A photograph to show a single RSF (with the weight of 7 mg and the length of 15 cm) can hold up a 200 g weight without breaking. **b**–**d** Tensile stress-strain curves (**b**), modulus (**c**), and toughness (**d**) of RSFs in experiments and simulations. The *error bars* in **c** and **d** were calculated as a s.d. The mechanical properties of RSFs obtained from experiments are divided into five categories according to their CSAs, which yielded by different reeling speeds. The relationship between CSA and reeling speed are summarized in Supplementary Table 2. Sort 1: CSA, 0.002 ± 0.001 mm^2^; Sort 2: CSA, 0.007 ± 0.001 mm^2^; Sort 3: CSA 0.014 ± 0.003 mm^2^; Sort 4: CSA, 0.018 ± 0.002 mm^2^; Sort 5: CSA, 0.024 ± 0.003 mm^2^. **e** Comparison of Young’s modulus and densities of RSFs with other materials. The Ashby plot was redrawn from refs.^46–48^. **f** Schematics of the computational model of a RSF in tensile test. We modeled a unit section of RSF as a bundle of SMFs of 1000 µm and simulated its mechanical behavior in tension. The RSF thickness played an important role in governing failure, as the critical cross-section area *A*_c_ = 0.008 mm^2^ governed the transition from brittle to ductile failure according to the simulations. **g** Simulation snapshots of two RSFs of different cross-section areas (*A*) in tension that fail in different ways. **h** The stress-strain curve of pristine RSFs of different cross-section areas obtained in computational simulations, with the curves of the defected samples summarized in Supplementary Fig. 4

Since the RSFs retain the structural hierarchy and well-organized silk nanofibril structures of natural silks (Fig. 1e and *inset* of Fig. 2i), which is critical for enhanced strength, extensibility, and toughness of silk fibers^24^, the RSFs exhibit high mechanical performance, defined as mechanical properties with strength, extensibility, and modulus equal or higher than 100 MPa, 20% and 5 GPa, respectively. These values are determined based on the mechanical performance of previously reported RSFs, which are listed in Supplementary Table 1. A single 7 mg RSF with length of 15 cm, as an example, can hold a 200 g weight without breaking (Fig. 3a). Tensile tests were carried out to measure the specific mechanical properties of the RSFs prepared by reeling (details can be found in “Methods”). A strong correlation between reeling speed and cross-sectional area (CSA) of the RSFs was observed in which the average CSAs of RSFs varied from 0.024 ± 0.003 to 0.002 ± 0.001 mm^2^ with reeling speeds from 4 to 14 mm s^−1^ (Supplementary Table 2). Accordingly, the mechanical properties of RSFs are divided into five categories according to their average CSAs, due to the changing reeling speed (Fig. 3b–d, Supplementary Table 2). Although these RSFs have a variation in mechanical properties in each category (particularly for strain to break), a direct correlation between CSAs and mechanical properties of the RSFs can be observed. By progressively increasing the CSA from 0.002 ± 0.001 mm^2^ (1^st^ sort; reeling speed: 14 mm s^−1^) to 0.024 ± 0.003 mm^2^ (5^th^ sort; reeling speed: 4 mm s^−1^), the tensile modulus of RSFs decreased from 11 ± 4 to 8 ± 1 GPa (Fig. 3c), while the toughness increased from 2 ± 2 to14 ± 9 MJ m^−3^ (Fig. 3d). The minimum average modulus of RSFs is 8 ± 1 GPa (5^th^ sort) (Fig. 3c), which is significantly higher than other values reported for as-spun RSFs from spider and *B. mori* silkworm silk fibroin (Supplementary Table 1) and comparable with natural *B. mori* silkworm cocoon silks (7 GPa^11^). The maximum modulus of RSFs (1^st^ sort, 11 ± 4 GPa, the highest value can reach up to 19 GPa) was even higher than that of *Araneus* major ampullate gland silk (10 GPa^1^) and most other natural biomaterials (Fig. 3e)^46–48^. In forced reeled animal silk fibers, besides impacting CSAs, the reeling speed also has a significant effect on the structure of the resultant fibers, where the higher reeling speed resulted in higher crystallinity and higher molecular aligement^49–55^. The same tendency was also observed in RSFs, for example, compared with sort 5 with the lowest drawing speed, sort 1 with the highest reeling speed presented increased crystallinity and molecular alignment that was confirmed by FTIR. By increasing the reeling speed from 4 to 14 mm s^−1^, β-sheet content increased gradually from 34 ± 5 to 45 ± 3% (Supplementary Fig. 3 and Supplementary Table 2). Furthermore, compared with RSFs reeled at 4 mm s^−1^ (5^th^ sort), RSFs reeled at 14 mm s^−1^ (1^st^ sort) showed more significant FTIR dichroism (Supplementary Fig. 3d), indicating higher molecular alignment. As a result, sort 5 has higher toughness but lower modulus than sort 1, because the modulus of silks is determined by the crystallinity and alignment, while the tensile strain is impacted by the amorphous regions^29, 56^.

In addition, the size effect influences the mechanical behavior of the RSFs. It has been shown in the former study that the diameter of the spider silk fiber plays a crucial role in affecting the fracture mode and toughness modulus of the fibers at the small size scale^24, 26^, because of the interplay of β-sheet nanocrystals and semiamorphous protein domains. However, compared to the critical thickness value of ${H^*}$= 22 nm^24^, our thinnest RSF with diameter of *H* = 70 µm is far from the scale region that will be affected by such nanoscale size effects, ($\sqrt {{H^*}/H} \ll 0.5$). Therefore, the size effect as discovered in the experiments in the current study is caused by mechanisms at the mesoscopic level. A computational model based on elastic network features was used to quantify and explain how RSF toughness increased with diameter. The computational models (Fig. 3f) are composed of a bundle of SMFs with each explicitly modeled, which allowed us to simulate RSF deformation up to mechanical failure. Considering the fact that different diameters of RSFs are the result of different reeling speeds, which yield different shear stress in spinning, this leads to differences in silk assembly because of the protein structural transitions and the formation of hydrogen bonds during stress^57, 58^. We effectively modeled the interaction strength between neighboring SMFs, which was inversely proportional to the RSF diameter, according to a former study^59^. The stress-strain curves were recorded for each of the pristine RSFs (Fig. 3h) and defect samples (with 0.1–0.5% defect rate, Supplementary Fig. 4), and statistically summarize the materials’ Young’s modulus and material toughness as the integration of area below the stress-strain curve (Fig. 3c, d), respectively. The simulation results showed that increasing RSF diameter leads to a smaller Young’s modulus but higher toughness, as the modulus and toughness changed from 11.2 ± 0.2 GPa and 3.3 ± 0.7 MJ m^−3^ for RSF of 0.002 mm^2^ to 8.0 ± 1.1 GPa and 14.2 ± 2.4 MJ m^−3^ for RSF of 0.025 mm^2^ in cross-section area, respectively (Fig. 3c, d). Good agreement was found between computational simulation results and experiment. More importantly, the mechanism is clearly shown as a thin RSF fails by breaking all the SMFs at a single point, leading to brittle failure, while thick RSFs, because of the low inter-fiber interactions, break by having fibers slide against each other, leading to ductile failure, as supported by simulation snapshots in Fig. 3g (necking of RSF and sliding between SMFs) and schematics in Fig. 3f.

**Fig. 4:**
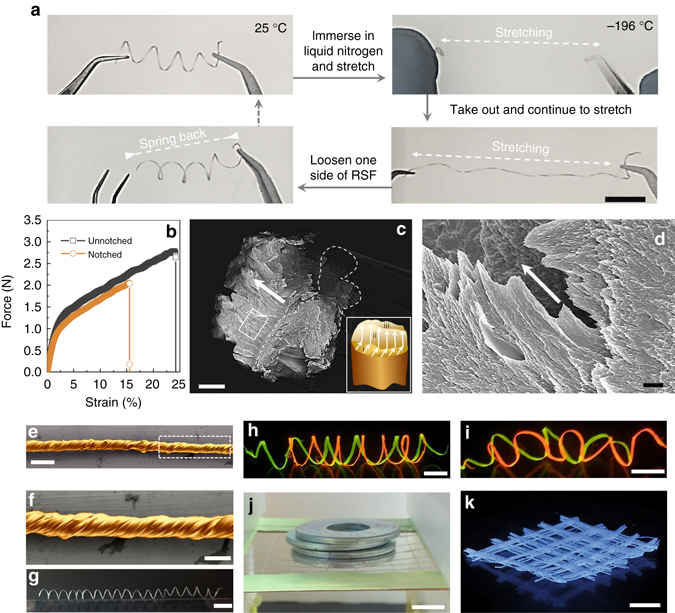
Polymorphic hierarchical RSFs produced by bioinspired spinning and their physical properties. **a** Mechanical performance of RSF under ultra-low temperature. The RSFs retained flexibility after immersion in liquid nitrogen. **b** Load-strain curves of notched and unnotched RSFs. More details about the notching experiments can be found in Supplementary Fig. 6. **c** Cross-sectional SEM image of notched RSFs after tensile fracture, which reveals three distinct regions (i–iii), as shown in the *inset*. The regions i, ii, and iii are a notch, crack stable growth area, and crack unstable growth area, respectively. **d** Locally amplified SEM image from *white solid frame* region of Fig. 5c. The *white row* shows the tensile direction. The image shows nanofibrils pulled out along the drawing direction. **e**–**k** Polymorphic architectures of RSFs prepared from bioinspired spinning. **e**, **f** SEM image of a yarn-like spiral RSF, produced by rotating the collector in a plane direction that is perpendicular to the fiber axis. Image **f** is the amplified SEM image from *white dashed frame region* of image **e**. False color is used in SEM images. **g** Photograph of a free-standing gourd vine-like helix RSF. **h**, **i** Photographs of colored luminescent RSFs under UV light with parallel (**h**) and cross-double helical (**i**) construction. The *red* and *yellow* color of RSFs is by adding Rhodamine B and Rhodamine 123, respectively. **j**, **k** Photographs of RSF-based 2D and 3D structures, fabricated from the bioinspired spinning process. *Scale bars*, 2 cm (**a**), 50 µm (**c**), 1 µm (**d**), 200 µm (**e**), 100 µm (**f**), 2 cm (**g**), 1 cm (**h**–**k**)

Structural hierarchy endows natural silks with fascinating physical properties. A typical example is the ultra-low temperature toughness of silks; silk fibers exhibit ductile failure even at the temperature of liquid nitrogen (−196 °C), and breaking elongation does not differ from the behavior seen at room temperature^60, 61^. Another example is the unique fracture mode and tensile behavior of notched silks; crack direction derives from the notch can be deflected to fiber longitudinal direction due to the longitudinally arranged silk nanofibrils in silks^62^. The RSFs reserve the structural hierarchy of natural silks, so we further evaluate the fracture behavior and ultra-low temperature mechanical performance of RSFs. To estimate the flexibility of RSFs in ultra-low temperature, a helical fiber was immersed in liquid nitrogen and then stretched to uncoil the helical structures (Fig. 4a and Supplementary Movie 1). The fiber is resilient (recoiled) immediately after being taken out of the liquid nitrogen. In contrast, other materials that are flexible at room temperature, such as cellulose paper and nitrile rubber, loose elasticity or break during immersing in liquid nitrogen (Supplementary Fig. 5 and Supplementary Movies 2, 3). The same fracture mode with natural silks is also observed in RSFs (Fig. 4b–d). In these experiments, an artificial notch was introduced in RSF, and the mechanical properties were tested to compare with that of the adjacent intact (un-notched) fiber (Supplementary Fig. 6). The notched RSF exhibits the same load-strain curve as the un-notched RSF (Fig. 4b); only the strain is reduced. This mechanical feature is the typical ductile fracture behavior of natural silks^62^. Cross-sectional SEM image (Fig. 4c) of notched RSFs after tensile fracture confirms the ductile fracture behavior. Three distinct fracture regions are shown (i–iii, as shown in *inset* scheme of Fig. 4c): the notched area (region i), the crack stable growth area (region ii), and the crack unstable growth area (region iii). The locally amplified SEM image (Fig. 4d) in the crack stable growth area reveals that the silk nanofibrils pulled out along the tensile direction after fracture. As with native silks, the crack growth direction is deflected from the fiber cross-section direction to the longitudinal direction.

### Polymorphic RSFs

As in natural spinning, a unique advantage of our bioinspired spinning system is to directly build 1–3 dimensional structures (Fig. 4e–k) during spinning without additional processes. Therefore, this bioinspired spinning approach provides an approach to utilize silks to generate polymorphic hierarchical RSFs with useful structures beyond mimicking fiber construction. For instance, a yarn-like spiral fiber can be produced by rotating the collector in a plane direction perpendicular to the fiber axis (Fig. 4e, f and Supplementary Fig. 7); a freestanding *Towel Gourd* tendril-like helix fibers^63^ are generated by extruding the spinning solution onto a cylindrical collector (Fig. 4g and Supplementary Fig. 8a, b). In addition, the silk fibrils in the spinning solution have the ability to absorb different types of dyes^44, 64^, thus suitable for generating colored fibers, which have shown promising applications in fashion, optical devices, and biomedicine^65, 66^. Figure 4h and i give examples of two specimens in which multicolored luminescent RSFs with parallel and cross-double helical construction were built by adding Rhodamine B and Rhodamine 123, respectively. More complicated 2D and 3D structures can also be generated, such as robust webs and grids (Fig. 4j, k and Supplementary Fig. 8c, d). The use of the hierarchical RSFs for biomedical applications was assessed by seeding human dermal fibroblasts (HDFs) on yarn-like spiral and as-spun RSFs. Cells grew along the fiber axes and 3D cell patterns following the contour of the RSF templates were generated on RSFs of different hierarchical structures (Supplementary Fig. 9a–d). Such macroscopically aligned constructs may be suitable templates to generate highly aligned tissues, such as muscle fibers, spinal cord, and tendon. Moreover, cells formed confluent cell layer on both type of RSFs by day 7 (Supplementary Fig. 9e, f), suggesting cell compatibility.

### Design and fabrication of conductive RSFs for monitoring the humidity and temperature

**Fig. 5:**
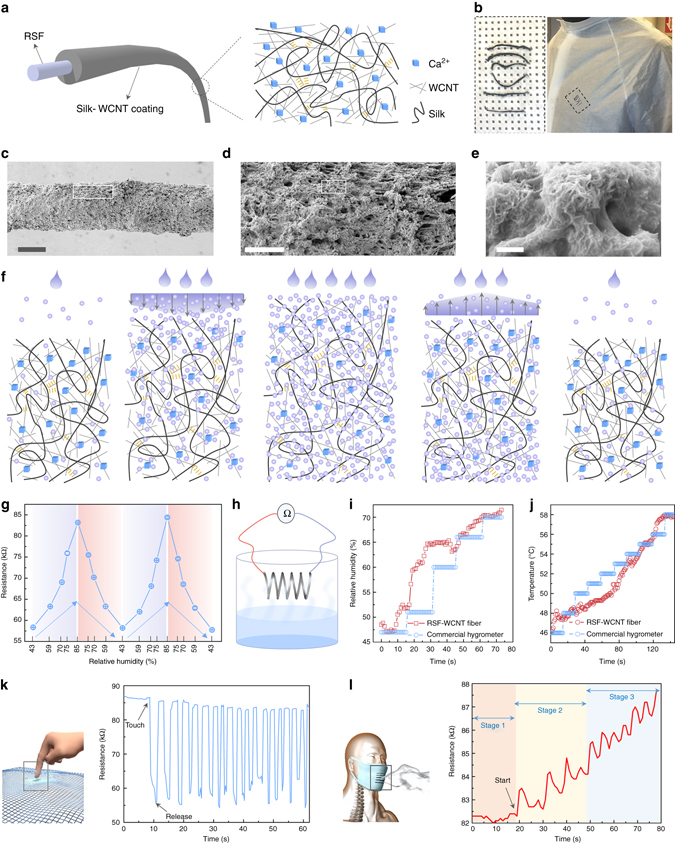
Examples of functional RSFs by dip-coating a conductive layer. **a** Illustration of the constitution of conductive core–shell RSFs. The core and coat layer are composed of RSF and silk/WMCNT/Ca^2+^ hybrid composites, respectively. **b** A conductive RSF is weaved into a cloth. These photographs show conductive RSFs are robust and can be weaved to different patterns. **c**–**e** SEM images of conductive RSF surfaces at different magnifications. Scale bars, 200 µm (**c**), 50 µm (**d**), and 500 nm (**e**). The coating layer shows a porous structure, which increased specific surface area and conducive to absorbing more water. The closely stacked WMCNTs, as a conductive composite, observed on the coating layer. **f** Schematic of humidity response mechanism of conductive RSFs. **g** The relationship between RH and resistance. The *error bars* in **g** were calculated as a s.d. of at least three measurements. **h** Schematic of experimental setups for monitoring humidity and temperature responses of conductive RSFs. **i** Time-resolved resistance vs. RH results of RSF sensor and commercial RH sensor. **j** Time-resolved resistance vs. temperature results of RSF sensor and commercial temperature sensor. In order to avoid RH effects on resistance, the RH is kept at 20% during the measurements. **k**, **l** Resistance response for finger-touching (**k**) and breathing (**l**)

The utility of these RSFs can be expanded by incorporating inorganic functional components. For example, the RSFs can be used to construct wearable humidity and temperature sensors via a three-step dip-coating method^67–69^, for which core–shell-based conductive fibers are used because they are easy to implement and maintain the excellent mechanical properties of RSF (Fig. 5). Briefly, multi-wall carbon nanotubes (MWCNT) were dispersed in formic acid/Ca^2+^ with 1 h sonication, followed by dissolving the degummed silk fibers in this solution with intense shaking. Then, the WMCNT/silk/Ca^2+^ ink was coated onto RSFs and dried at room temperature to eliminate the formic acid (Fig. 5a, b). The conductive coating layer closely bonds with the RSFs (Fig. 5c–e and Supplementary Fig. 10) since the formic acid/Ca^2+^ solvent system dissolve the surface of RSFs^64^. More significantly, the Ca^2+^ ions in the coating layer capture water from the environment through coordination complexes; a Ca^2+^ ion can coordinate 6–8 water molecules via the oxygen atoms^70, 71^. As shown in Fig. 5f, the higher the relative humidity (RH) in the environment, the more the water that can be captured in the coating layer. Therefore, the coating layer gradually swells and the distance between WMCNTs widens progressively with the increase of RH. These processes are reversible. Once the RH is reduced to the initial value, the coating layer dimension and WMCNT distances recover to their starting states. As a result, the resistance of WMCNT/silk/Ca^2+^ coatings is very sensitive to humidity changes (Fig. 5g–i).

Figure 5g reveals the relationship between RH and resistance. When the RH increases from 43 to 85%, the resistance increase gradually from 58.4 ± 0.1 to 83.2 ± 0.1 kΩ. After four cycles, the resistances are similar at the repeated same RH, demonstrating the reversibility of the process. A time-resolved resistance vs. RH experiment (Fig. 5h and Supplementary Fig. 11) was designed to evaluate the response rate related to the change in RH. A conductive RSF was fixed on the top of a 2 L glass bottle, then the ~50 ml 50 °C water was added to the bottom of the bottle. The temperature of RSF position was kept at 24–25 °C and no changes were detected during the test process. We find that the resistance of the RSF increases after 4 s by adding the water to the bottle (Fig. 5i). Considering the diffusion rate of water vapor, the resistance is synchronous in ascending with the increase of RH, and more rapidly than a commercial hygrometer (15 s). Significantly, the resistance of conductive RSF varies in this time-resolved process, and is more sensitive to RH changes than a commercial hygrometer, which offers a step-wise response. The conductive RSF also quickly responses to changing temperature (Fig. 5j); the resistance of RSF decreases with increased temperature. After standardization of the resistance to initial temperature, the plot coincides with recordings from a commercial thermometer. These rapid responses to humidity suggest that these conductive RSFs could be utilized in clothes and masks toward smart fabrics to sense and monitor touching and breathing. As presented in Fig. 5k, l, the resistances respond real-timely upon touching the cloth with a finger (Fig. 5k) or breathing (Fig. 5l), and rapidly recover to the original state once the stimulus was removed. These RH and temperature-sensitive wearable SNFs may find applications in wearable sensors, considering the biocompatible nature of the composites, even in medical implants.

## Discussion

Silks, as one of the most abundant natural polymers (biopolymers) on earth, have attracted intense attention in recent decades due to their renewability, wide availability, biocompatibility, and biodegradability^1–3^. A variety of processing methods have been developed to generate silk materials with different formats, but in most cases, their mechanical properties are much weaker than that of natural silks^3^. This gap drives us to rethink the conventional protocols of silk processing, especially when we mainly focus on the mechanical and functional performance of the designed materials. Directly utilizing native silks and/or silk building blocks to create structural and functional materials will be an optimal choice, because these ways can maximum effectively retain advantages of natural materials.

As a prototype illustrated in this study, we dissolved the silk fibers into microfibrils instead of thoroughly into silk fibroin molecules. These microfibrils maintained sophisticated nano-architecture and micro-architecture in silks and meanwhile can be spun into polymorphic RSFs through the bioinspired spinning process. With the advantages of natural silks, these as-spun RSFs indeed show outstanding mechanical properties, which are comparable to and even superior to natural silks. In addition, similar to 3D printing, our bioinspired spinning process can be used to build sophisticated 1D–3D structures. This method provides a useful approach to generate functional materials, such as textiles, surgical sutures, and tissue scaffolds.

This spinning process is also similar to natural fiber welding^72–78^, a process where loose fibers are transformed to create a congealed network using an ionic liquid. Both methods first involve the partial dissolution of the natural fibers, which are then processed into different materials. The mechanism of natural fiber welding is to dissolve the outer portion of individual natural fibers (i.e., cellulose, hemicelluloses, silk) and then the fibers can be bonded together by the dissolved polymers. In the whole natural fiber welding process, the fibers maintain their original length and appear as solid fibers, so the process is suitable for building largescale bulk materials. Our spinning strategy is more like a microscale natural fiber welding process. The silk fibers are partially dissolved and cut into microfibrils by HFIP, the resultant system is a viscous microfibril solution with nematic texture, thus able to be spun out continually to form microscale RSFs and also can be processed into other microscale formats. Both methods allow the formation of ordered constructions without destroying the structural and mechanical features of the natural fibers. More remarkably, the applications of these constructions can be widened, for example, RSFs can be coated with a conductive layer. The resultant conductive RSFs can be woven into textiles to make wearable RH and temperature sensors. These low-cost, conductive RSFs may be useful in wearable sensors, biosensors, and implant devices. We believe these attempts, directly using natural materials, could help to create functional composites with high mechanical performance.

## Methods

### Preparation of SMF spinning dope

*B. mori* silkworm cocoon silk fibers were degummed by boiling in two 30 min changes of 0.5% (w/w) NaHCO_3_ (Sigma-Aldrich, USA) solution^79, 80^. The degummed silk fibers were washed with distilled water and allowed to air dry at room temperature. The degummed *B. mori* silk fibers were then immersed in HFIP solution with a weight ratio of 1:20, and sufficient oscillation was applied so that all fibers were immersed. The SMF spinning dope was obtained after incubating airtight containers with the silk fiber/HFIP mixture at 60 °C for 7–15 days. Because the HFIP is a toxic solvent, all of these steps should be conducted in a chemical hood with the necessary precautions.

### Bioinspired spinning methods

The SMF dope was transferred to a syringe with a needle (needle gauge: 25 mm; the inner diameter: 0.8 mm), then the dope was directly spun from the syringe needle at room temperature. During the spinning process, three approaches were used to apply shear force to the spinning dope. (1) Syringe pump extrusion method. The helical *Towel Gourd* tendril-like RSFs were prepared by this method. In this process, the SMF dope was directly extruded onto a polytetrafluoroethylene (PTFE) guide roll (diameter of 10 mm) via a syringe pump (New Era Pump Systems NE-1800, USA) with a flow rate of 20 ml h^−1^. During the spinning process, the needle tip was fixed and contacted with the top of the PTFE roll, and the PTFE roll was manually rotated (angular speed: ~0.785 rad s^−1^) and moved gradually along its *z*-axis (moving speed: ~1 mm s^−1^). (2) Manual extrusion method. The yarn-like spiral RSFs and 2D/3D constructions of RSFs were spun by this method. Specifically, in yarn-like spiral fiber spinning process, the initial as-spun RSF was fixed onto a collector (i.e., cardboard, glass, and plastic films) using double sticky tape and then the collector was rotated in a plane direction perpendicular to the fiber long axis. Meanwhile, the collector was moved continuously to the far end (the direction away from the needle tip) with a speed around 1 mm s^−1^. In terms of spinning 2D and 3D structures, the SMF dope was directly spun onto a cardboard frame with a hollow size of 60 mm × 60 mm (2D web) or a PTFE substrate (3D structures) along the warp and weft directions. The spacing between the fibers in the warp and weft direction was fixed at 7 mm. (3) Forced reeling approach. RSFs used for mechanical tests were prepared by this method. A small-scale custom-build roller-type reeling apparatus was used to collect the RSFs. The roller was made of polypropylene with a diameter and length of 30 and 100 mm, respectively. The rotating speed of roller was controlled by an electronic system composed of a gear box motor and an electronic controller. Before the reeling, the roller speed was set up in advance and the dope syringe was fixed to the left side of the roller. The needle tip was parallel to the top of the roller with a separation distance of 50 mm. With the start of winding, the dope was pulled out from needle tip and fixed on the top of the roller with the help of sharp stainless tweezers and then the motor was switched on to reel the RSFs. All of the above methods were conducted in a chemical fume hood with necessary precautions.

### Preparation of conductive silk/carbon nanotube solution

One gram CaCl_2_ (Sigma-Aldrich, USA) was added in 20 g formic acid (Sigma-Aldrich, USA) solution, followed by the addition of 100 mg MWCNT (Sigma-Aldrich, USA). After ultrasonication for 1 h at room temperature, 1 g degummed silk fiber was added in solution with intense shaking to obtain the conductive silk/MWCNT solution. All steps are required to be conducted in a chemical fume hood with the necessary protection.

### Mechanical testing

First, a 50 mm RSF was cut into two RSF segments with lengths of 20 and 30 mm, respectively. The 20 mm RSF segment was used to measure the CSA, and the 30 mm RSF segment for the tensile test. For tensile testing, the 30 mm RSF segment was mounted on a hard cardboard frame with a base length of 10 mm and fixed with cyanoacrylate. After the cyanoacrylate was totally dried overnight, the frame was mounted in the testing machine and the side support of the frame was cut away so that the force was transmitted through the RSF. Meanwhile, the initial length of the fiber was measured with a caliper at zero load point (the point in which the RSF is tight but no force exerted on it). The mechanical tests were carried out by using an Instron 3366 machine (Instron, Norwood, USA) in tensile mode at 25 °C and 50% RH with a tensile speed of 0.5 mm min^−1^. In terms of CSA measurement, the 20 mm RSF was fixed with cyanoacrylate, and after drying overnight the samples were sectioned into three segments in liquid nitrogen directly using fresh razor blades. Then these three samples were mounted onto SEM stubs and sputter coated with a 5-nm-thick Pd/Pt layer. The cross-sections were observed by SEM (Ultra 55 field emission scanning electron microscope, Carl Zeiss AG, Harvard University Center for Nanoscale Systems). The smooth cross-sections of RSF segments were observed and the CSA was estimated by ImageJ software (NIH). The average area of three segments was used as the CSA of adjacent RSFs and then used for stress calculations.

### FTIR spectroscopy measurements

The structures of degummed silk fibers and RSFs with different reeling speeds were characterized by FTIR spectroscopy in ATR mode (Jasco FTIR-6200, Jasco Instruments, Easton, MD). For each measurement, 64 interferograms were co-added and Fourier-transformed employed a Genzel-Happ apodization function to yield spectra with a nominal resolution of 4 cm^−1^. Deconvolution of amide I bands was carried out using PeakFit 4.12^79–81^. The numbers and positions of peaks were defined from the results of second derivative spectra and fixed during the deconvolution process. A Gaussian model was selected for the band shape and the bandwidth, which was automatically adjusted by the software. It should be noted that each spectrum shown was from a single experiment, but the data obtained from the spectra (e.g., β-sheet content) were the average of five separate deconvolutions from different samples. FTIR dichroism has been widely used to determine the molecular orientation in silk fibers, using the dichroism of amide bands^81, 82^. The spectra were recorded in ATR mode with infrared red light polarized either parallel or vertical to the long axis of the fiber. All spectra were normalized by the intensity at 1450 cm^−1^ assigned to anti-symmetric bending of the methyl group. This band was used as it is insensitive to the conformation of silks^81, 82^.

### Computational simulations

A RSF was modeled by a bundle of SMFs with each of them modeled by a 1D coarse-grained elastic network composed of a series of mass beads (with *m* = 5.5 × 10^−10^ g for each bead, corresponding to the density of silk) connected by nonlinear elastic springs, as we have used to simulate the mechanics of a spider web^83^. The equilibrium length of a spring, which is the same as the inter-bead distance of the neighboring beads of the initial model, was of *r*_0_ = 7.1 µm to define the coordinates of each mass bead in the SMF. The interaction between the two nearest neighboring mass beads was modeled by a nonlinear elastic spring with the bond energy (*E*_*k*_) given by a Morse potential as:

1 $${E_k} = D \cdot {\left[ {1 - {{\rm e}^{ - {{\alpha }}\left( {r - {r_0}} \right)}}} \right]^2},$$

where *D* is the bond energy, *α* is the parameter control the stiffness of the bond, and *r* is the bond length in the simulation. By adjusting the numerical values of the parameters (*D*, *α*), different force-extension curves of the pristine silk fiber were generated. Referring to the test on the thinnest RSF in experiments, assuming all SMFs deform homogeneously in tension, the numerical values of *D* = 1.41 × 10^−8^ J and *α* = 2.14 × 10^6^ m^−1^ are generated that give the force-strain curve (Fig. 3h) with Young’s modulus of 11.2 GPa and toughness of 3.0 MJ/m^3^. The SMFs were arranged in the close-packed form as shown in Fig. 3f, with neighboring fibers separated by *σ* = 10 µm, as *σ* the diameter of a SMF. The bending stiffness of a SMF is modeled by an angular spring between two neighboring springs, with the spring stiffness ${K_B} = \frac{{E{I_t}}}{{2{r_0}}} = \frac{{E{\sigma ^4}}}{{128{r_0}}} = 1.2 \times {10^{ - 7}}{\rm{J}}$ and equilibrium angle of 180°.

The interaction between beads in different SMFs is modeled by a simple Lennard-Jones potential *E*_*lj*_, given by

2 $${E_{lj}} = 4\varepsilon \left( R \right) \cdot \left[ {{{\left( {\frac{\sigma }{r}} \right)}^{12}} - {{\left( {\frac{\sigma }{r}} \right)}^6}} \right]$$

with *ε* the interacting energy and *r* is the distance between beads. Here, we take *ε* as a function of RSF radius (*R*) by considering the fact that *R* value is the result of different reeling speeds, which yields different shearing stress in spinning and is given by

3 $$\varepsilon \left( R \right) = \left( {\frac{1}{R} - \frac{1}{{{R_2}}}} \right)\frac{{{\varepsilon _1} - {\varepsilon _2}}}{{\frac{1}{{{R_1}}} - \frac{1}{{{R_2}}}}} + {\varepsilon _2}$$

where *R*_1_ = 0.026 mm as the thinnest silk fiber and *ε*_1_ = 0.6 × 10^−8^ J that gives the inter-fiber interaction energy of unit area the same as the SMF toughness, *R*_2_ = 0.09 mm as the thickest silk fiber and *ε*_2_ = *ε*_1_/6, according to former study^59^, which shows the shearing deformation increases the silk inter-molecular connectivity by six times.

Each SMF in the model was modeled as 1000 µm in length, and only simulated the unit section of the RSF in tension (Fig. 3f). To allow sliding of SMFs, we fixed the left end of SMFs with odd index and applied force to the right end of SMFs with even index in the simulations. The effect of defects was considered by randomly deleting the constituting mass beads and associated springs in a RSF model before running simulations in tension. We simulated the mechanics of RSFs of different diameters by applying a constant strain rate of 10 s^−1^ with a time step of 5 × 10^−8^ s by running molecular dynamics simulations with LAMMPS code package^84^.

### Cell culture on RSFs

HDFs derived from the dermis of human newborn foreskin were a generous gift from Garlick lab at Tufts University and cells of passage 14 were used for the experiment. The cells were cultured in Dulbecco’s Modified Eagle Medium (Invitrogen) supplemented with 10% fetal bovine serum (Sigma-Aldrich), and 100 U ml^−1^ penicillin, 100 μg ml^−1^ streptomycin (Invitrogen) at 37 °C, 5% CO_2_. Silk fibers were sterilized in 70% ethanol and thoroughly rinsed in sterile distilled water. The fibers were incubated in growth medium with the aforementioned compositions for 24 h before cell seeding. Cells were seeded on fibers by incubating fibers in a cell suspension of 0.5 × 10^6^ cells ml^−1^, and then the fibers were transferred to fresh medium after 4 h. Cell medium was changed every 2 days. The viability of the HDFs on silk fibers was assessed by live/dead assay (Molecular Probes). The cells were incubated in medium containing 2 μM calcein-AM and 4 μM EthD-1 at 37 °C for 15 min. The stained RSF/cell constructs were then observed with a Keyence BZX710 fluorescent microscope (Keyence). The presence of dead cells was analyzed by careful examination of red channel images and differentiating signals belonging the cells and the underlying fibers.

### Characterization

The texture of the SMF solution and the orientation of RSFs were assessed by polarizing optical microscope (Olympus BX51-P, Japan). The morphology of RSFs was characterized by SEM (Ultra 55 field-emission scanning electron microscope, Carl Zeiss AG, Harvard University Center for Nanoscale Systems) at an acceleration voltage of 5 kV. To prevent electrical charging, all specimens were coated with a 5-nm-thick Pd/Pt layer before observation. The conductivities of conductive RSF-based materials were assessed using a Fluke 87 V Digital multimeter. Before the tests, the conductive RSF ends were firmly fixed to multimeter test leads. The conductive RSF length between two leads was fixed to 10 cm for all of the tests. To record the resistances of conductive RSFs at different RH, a conductive RSF was gradually incubated at various RH levels controlled by specific saturated salt solutions with known RH: K_2_CO_3_ (43%); NaBr (59%); KI (70%); NaCl (75%); and KCl (85%). In terms of finger-touching and breathing measurements, the conductive RSFs with the length of ~15 cm were woven into clothes and masks, and their ends firmly fixed to multimeter test leads. The whole processes of time-response measurements were recorded by video camera, the related time and resistance values were directly extracted from each frame of recorded video with a time resolution of ~0.3 s.

### Data availability

The authors declare that all data supporting the findings of this study are available within the article and its Supplementary Information Files or from the corresponding author on reasonable request.

## Electronic supplementary material

Supplementary Information

Supplementary Movie 1

Supplementary Movie 2

Supplementary Movie 3
