# Supplementary material for: Polymorphic regenerated silk fibers assembled through bioinspired spinning
Source: Nat Commun. 2017 Nov 9;8:1387. doi: 10.1038/s41467-017-00613-5 (PMC5680232; doi:10.1038/s41467-017-00613-5)
Supplement: Supplementary file 1 — Supplementary Information [file 41467_2017_613_MOESM1_ESM.pdf]

Description of Supplementary Files

File name: Supplementary Information
Description: Supplementary figures, supplementary tables and supplementary references.

File name: Supplementary Movie 1
Description: The flexibility of RSFs in ultra-low temperature.

File name: Supplementary Movie 2
Description: The flexibility of cellulose paper in ultra-low temperature.

File name: Supplementary Movie 3
Description: The flexibility of nitrile rubber in ultra-low temperature.

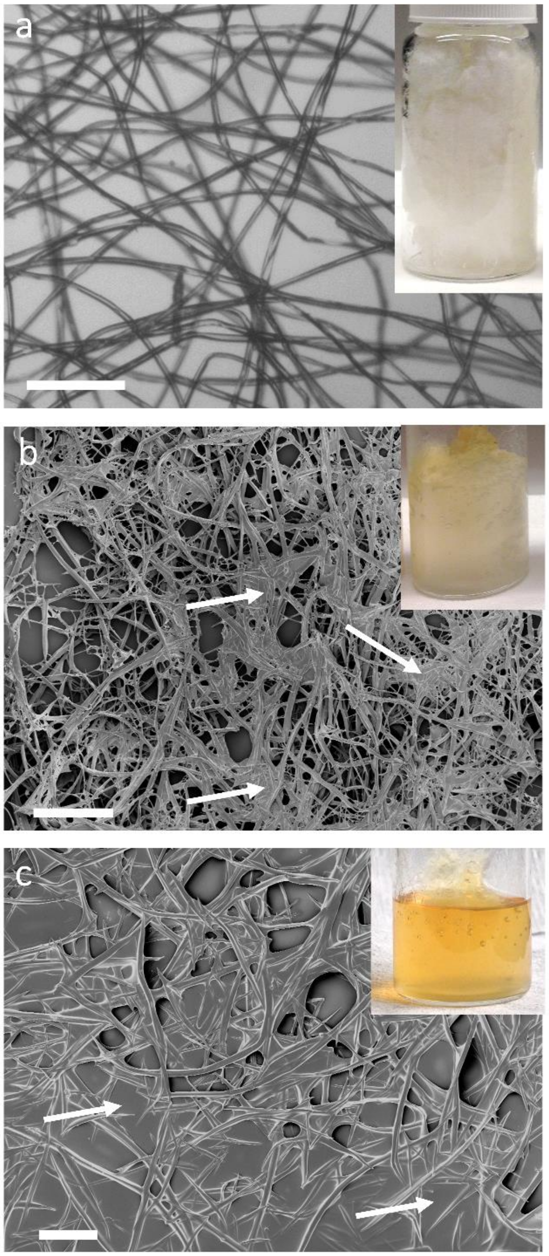

Supplementary Figure 1. The morphology changes of *Bombyx mori (B. mori)* silk fiber during the partial dissolution process. (**a**) Optical microscopy image of silk fiber after incubated in HFIP (weight ratio 1:20) at 60°C for 2 hours. The image shows that the silk fibers are still intact, and no cleavage is observed. (**b**) Scanning electron microscopy (SEM) image of *B. mori* silk fiber after incubated in HFIP (weight ratio 1:20) at 60°C for 4 days. These images show that *B. mori* silk fibers are dissolved and cut into shorter fibers. (**c**) SEM image of *B. mori* silk fiber after incubated in HFIP (weight ratio 1:20) at 60°C for 15 days. The image indicate that the fibers are partially dissolved to form the microfibrils. The white arrows in SEM images (b and c) shows the dissolved silk fibroin polymer. Scale bars, 200 µm (a,b) and 100 µm (c).

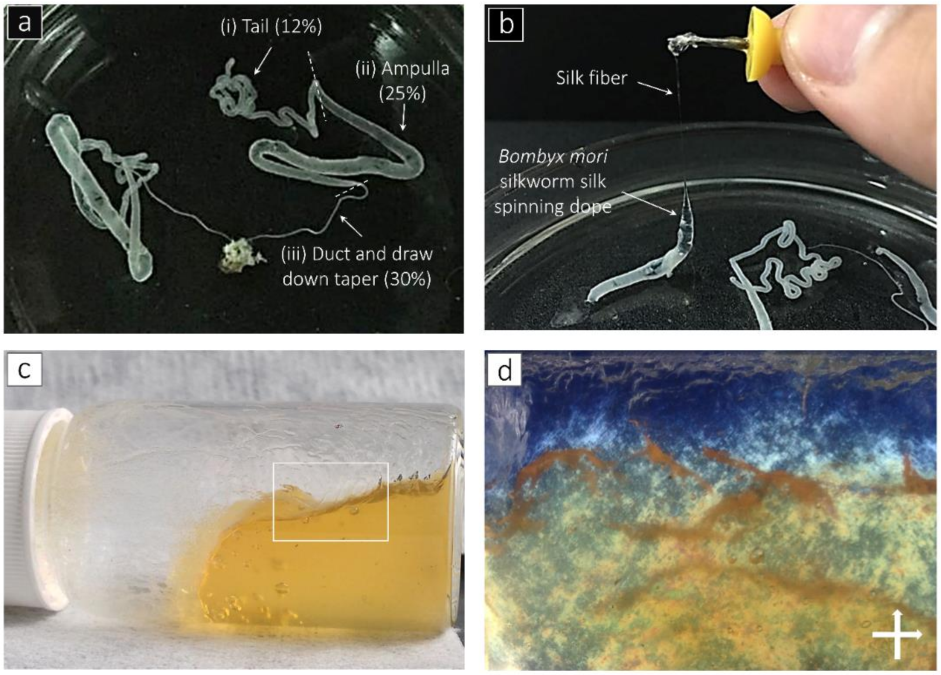

Supplementary Figure 2. (**a**) *B. mori* silkworm gland, (**b**) silk spinning dope. The pictures show that silk fibers can be directly formed by drawing the spinning dope. (**c,d**) the silk microfibril/hexafluoroisopropanol (SMF/HFIP) solution under visual (c) and polarized light (d).

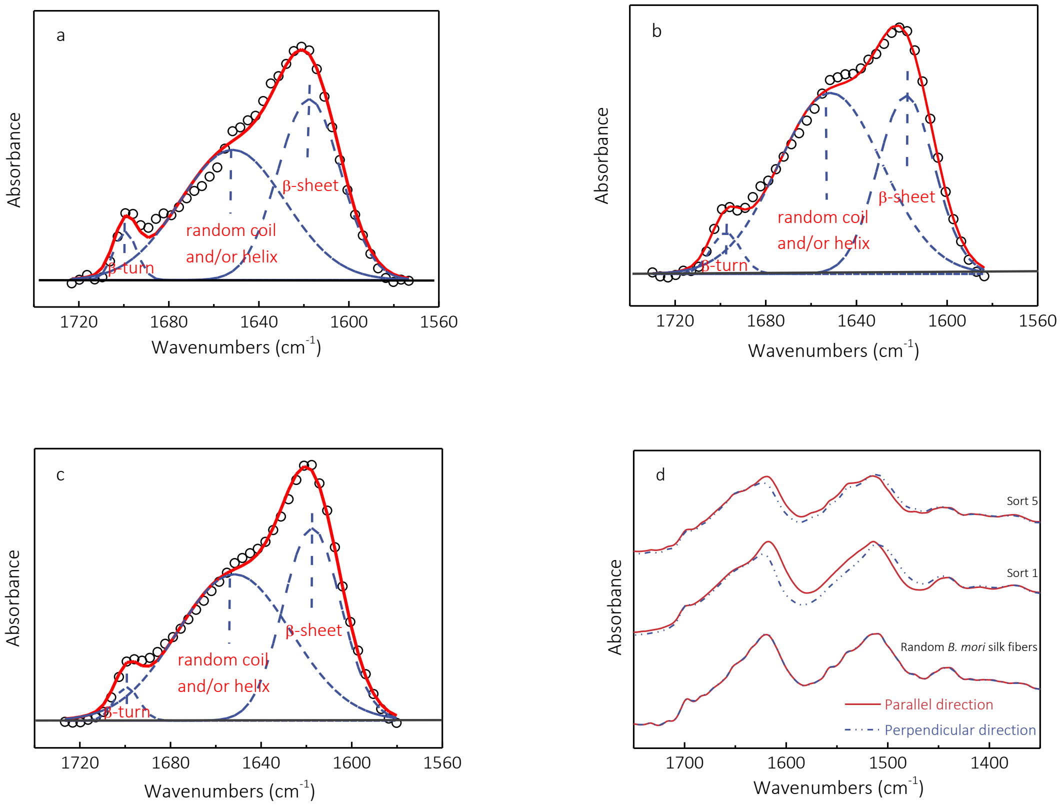

Supplementary Figure 3. FTIR spectra of RSFs and degummed *B. mori* silk fibers. (**a**) Deconvolution of the FTIR amide I band of RSFs with reeling speed of 14 mm s^{-1} (sort 1). (**b**) Deconvolution of the FTIR amide I band of RSFs with reeling speed of 4 mm s^{-1} (sort 5). (**c**) Deconvolution of the FTIR amide I band of degummed *B. mori* silk fibers. The FTIR spectra used for deconvolution were recorded by random aligned RSFs and degummed silk fibers. (**d**) Polarized FTIR spectra of RSFs with directional arrangement and degummed *B. mori* silk fibers with random arrangement. The red solid line is the fibers measured with the infrared beam parallel to the fiber axis; the blue dash-dot-dot line is the fibers measured with the infrared beam perpendicular to the fiber axis. Comapred with polarized FTIR spectra of random arranged *B. mori* silk fibers, the FTIR dichroism of the amide I band of RSFs confirmed the molecular alignment along the fiber axis. In addition, compared with sort 5 RSF samples (reeling speed: 4 mm s^{-1}) , sort 1 samples (reeling speed: 14 mm s^{-1}) showed more significant FTIR dichroism, indicting the sort 5 had higher molecular alignments.

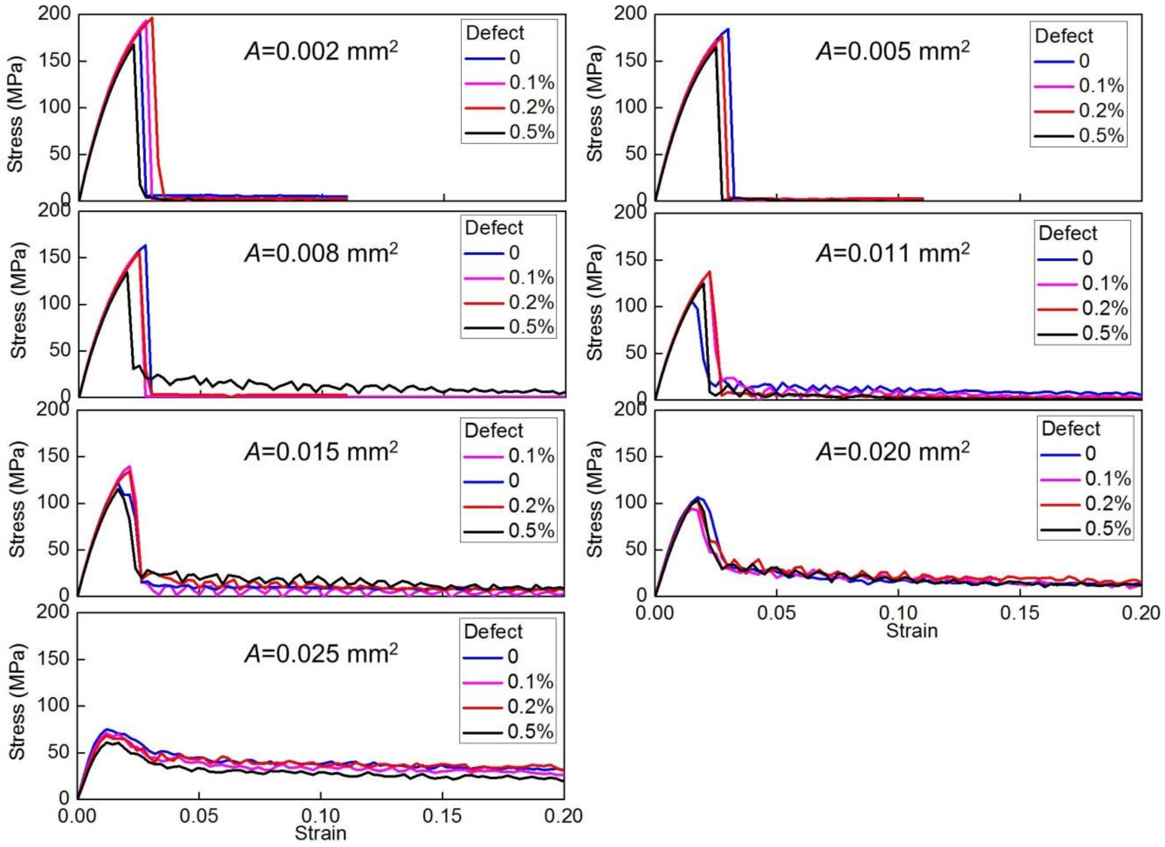

Supplementary Figure 4. The stress-strain curve of pristine and defected RSFs of different cross-section area (*A*) obtained from computational simulations.

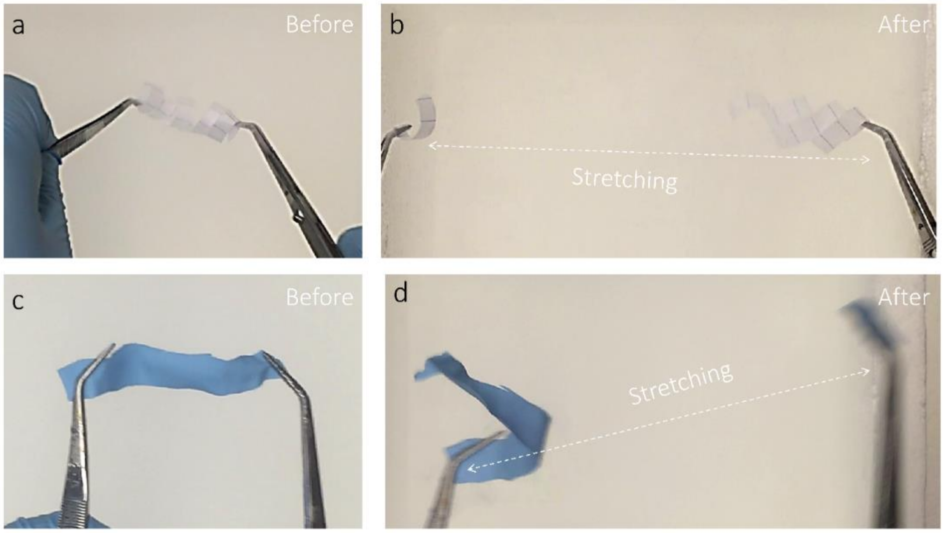

Supplementary Figure 5. (**a**) Cellulose paper before immersion in liquid nitrogen. (**b**) Cellulose paper undergoing stretching after immersion in liquid nitrogen. (**c**) Nitrile rubber film before immersion in liquid nitrogen. (**d**) Nitrile rubber film undergoing stretching after immersion in liquid nitrogen. These images indicate that the two materials are brittle at ultralow temperatures.

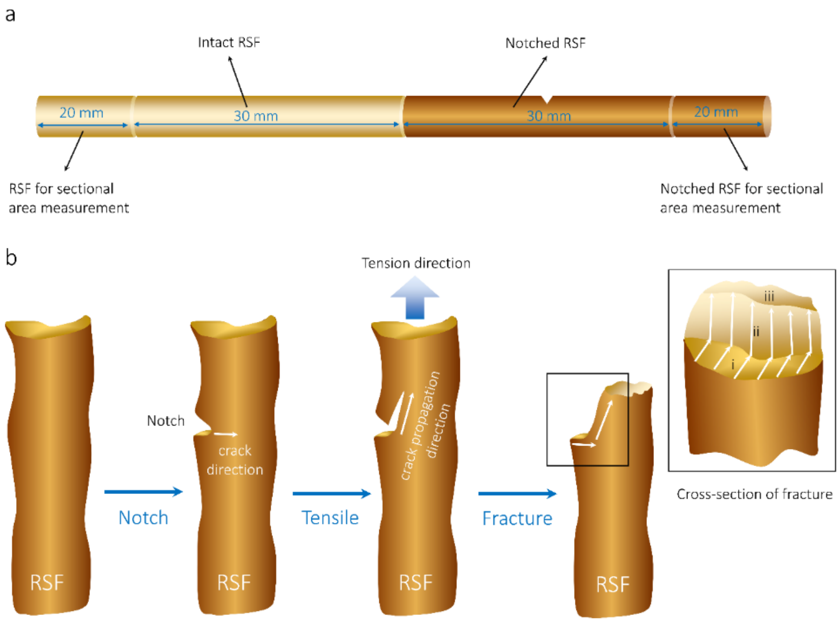

Supplementary Figure 6. (**a**) The arrangement of samples for different tests. The Figures and arrows indicate the length of the RSF used. To compare the mechanical properties of notched and un-notched RSFs, two adjacent segments were used. As shown in Figure (a), the right RSF segment was notched with a sharp scalpel from the edge with a depth of 50-100 μm. The notch is in the middle of fiber axis direction. Left RSF segments, without notching, were used for comparison. (**b**) Ductile fracture mechanisms of RSF. Unlike the brittle materials where the crack propagation direction was perpendicular to the direction of the fiber axis, the crack propagation direction was same as for the native silk fibers and shows three fracture regions. The region i, ii and iii are a notch, crack stable growth area, and crack unstable growth area, respectively.

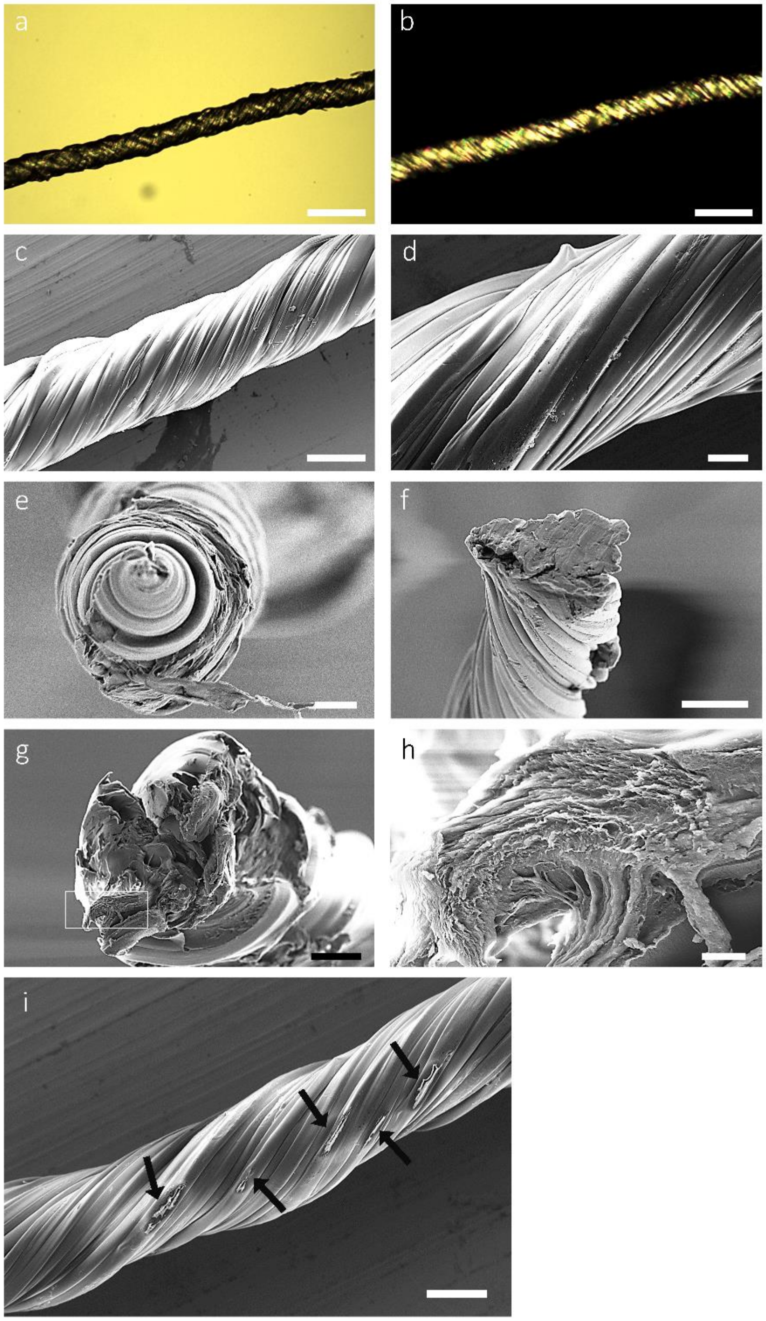

Supplementary Figure 7. SEM image of a yarn-like spiral RSF. (**a**) Microscopy image of yarn-like spiral RSF, (**b**) Polarized light microscopy of yarn-like spiral RSF, (**c,d**) Surface (c,d) and cross-sectional (**e,f**) SEM images of two types of yarn-like spiral RSFs with different pitches. (**g**) Cross-sectional SEM image of yarn-like RSF after tensile fracture. (**h**) the locally amplified SEM image from white solid frame region of figure g. The image (g) showed clear nanofibril structure in the RSF cross-section. (**i**) The defects of yarn-like spiral RSF, highlighted by black arrows. Scale bars, 200 µm (a,b), 50 µm (c, f, and i), 20 µm (d, e, g) and 2 µm (h).

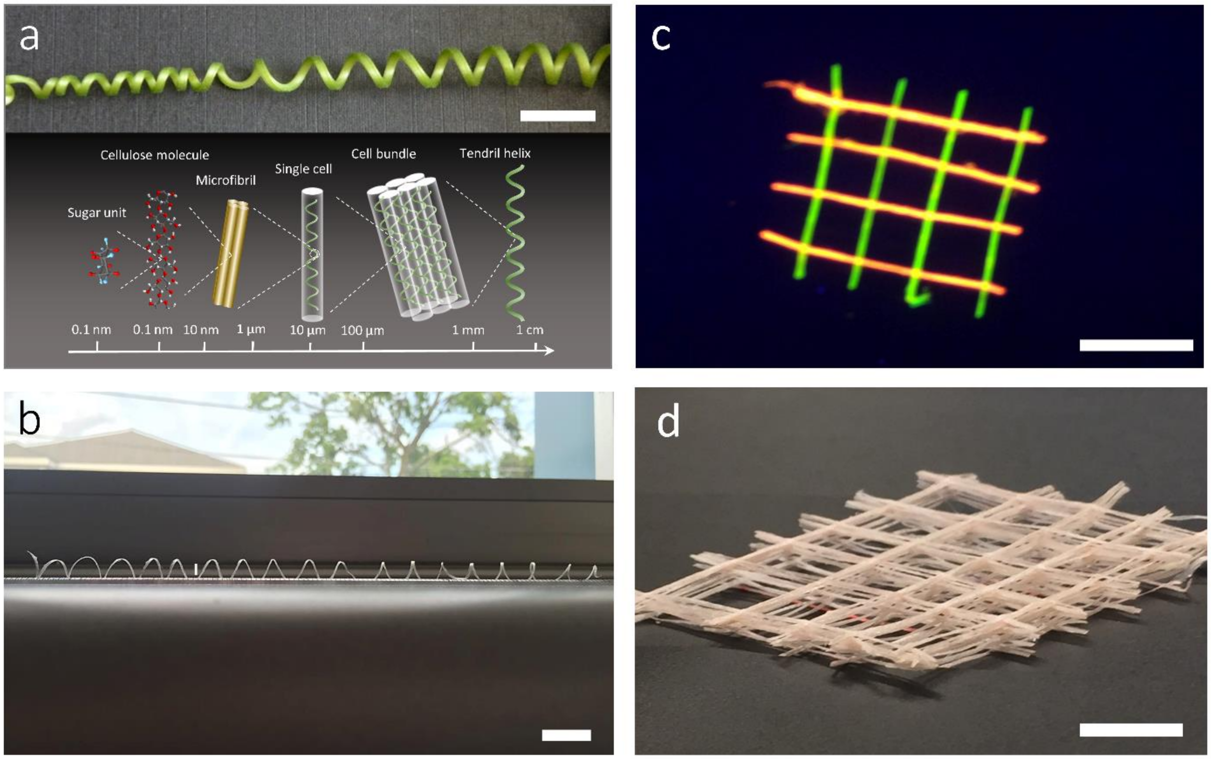

Supplementary Figure 8. (**a**) Hierarchical structures of helical gourd vine. The top and bottom images are the photograph of gourd vine and the schematic of the hierarchical structure of gourd vine, respectively. (**b**) Photograph of helical RSF, which showed the gourd vine-like structure. (**c**) Photograph of fluorescence-colored 2D RSF grid under UV light. The RSF fiber in warp and weft direction were spun by SMF dope containing Rhodamine B and Rhodamine 123, respectively. (**d**) Photograph of the free-standing 3D grid. Scale bars, 2 cm (b) and 1 cm (a, c and d).

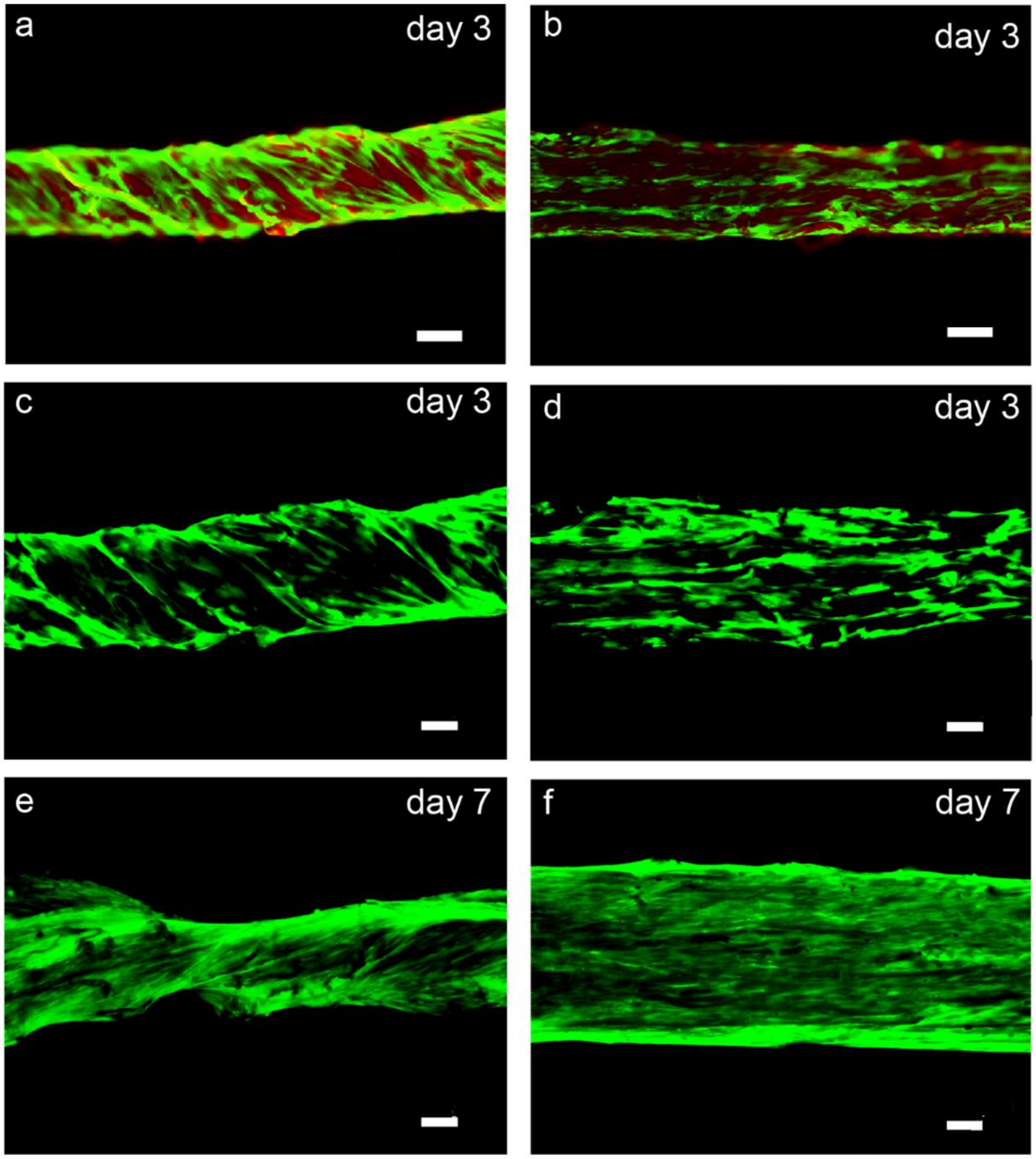

Supplementary Figure 9. Three-dimensional cell patterns generated on yarn-like spiral and as-spun RSFs. Cells were cultured for 3 and 7 days and cell viability assessed by live/dead staining. Fluorescent images showing the preferential alignment of HDFs (green) along the axes of the **a**, **c**, and **d**) yarn-like spiral and **b**, **d** and **f**) as-spun RSFs on day 3. Cells proliferated and formed a confluent layer on both types of RSFs by day 7. The silk fibers were stained uniformly red by EthD-1, which highlighted the topographical feature of RSFs (yarn-like spiral RSF and as-spun RSFs). The dead cells, whose nuclei stain bright red, were analyzed and no/minimal visible dead cells were detected. Scale bars, 200 μm (a,b) and 100 μm (c-f).

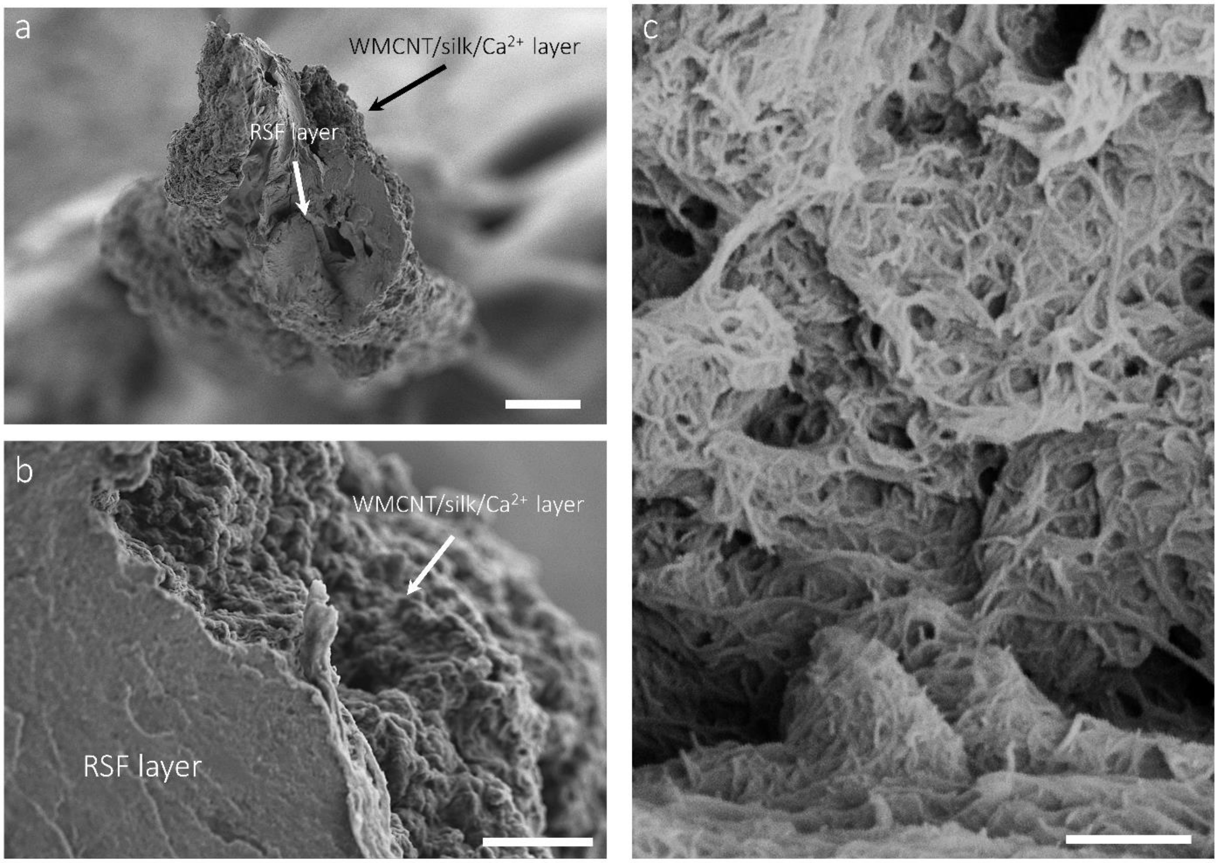

Supplementary Figure 10. SEM image of conductive RSFs. (**a,b**) Cross-sectional SEM image of conductive RSF under small (a) and high (b) magnification. These two images indicate that the WMCNT/silk/Ca^{2+} layers are bonded well with the RSF core. (**c**) Surface SEM image of conductive WMCNT/silk/Ca^{2+} layer. Scale bars, 100 µm (a), 10 µm (b) and 500 nm (c).

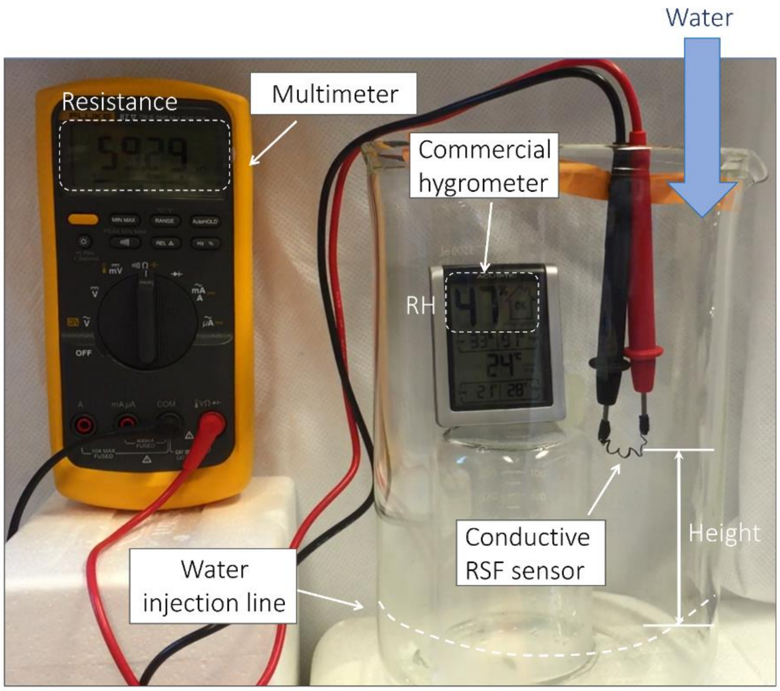

Supplementary Figure 11. Experimental setups for monitoring humidity and temperature response of conductive RSFs.

Supplementary Table 1. Comparison of mechanical properties of regenerated silk fibers.[a]

	Spinning dope[b]	Coagulation bath	Post-spin draw	Strength (MPa)	Extensibility (%)	Modulus (GPa)	Mechanical properties of as-spun fibers (stress; strain; modulus)[c]	Ref.
Bombyx mori RSF wet spinning	RSF/ $CaCl_2$/water, 15wt%	$(NH_4)_2SO_4$	9 (water)	314±19	37±4	10.4	U. C.	1
	RSF/water, 15% w/v	$(NH_4)_2SO_4$	6 (water)	450±20	27.7±4.2	12.5	Very weak	2
	RSF/water, 16% w/v	$(NH_4)_2SO_4$	4 (water)	390±50	32.1±5.8	15.2±3.3	Very weak	3
	RSF/water, U.C.	$(NH_4)_2SO_4$	No	2.5 g/den	20–25	U.C.	U.C.	4
	RSF/water, U.C.	$(NH_4)_2SO_4$, Na_2SO_4	No	2.1 g/den	10.1	U.C.	U.C.	5
	RSF/water, U.C.	$(NH_4)_2SO_4$	4 (water)	0.22	30±4	11.2	very weak	6
	RSF/water, 15 wt%	$(NH_4)_2SO_4$	9 (water)	450±30	27.3±4.6	18.9±1.1	U.C.	7
	RSF/water, 13 wt%	$(NH_4)_2SO_4$	4 (water)	98	58.9	37.8	U.C.	8
	RSF/PEG/LiBr	MeOH/Water	1.1	128.8	7.6	6	U.C.	9
	RSF/LiBr•H_2O–EtOH–H_2O, 20 wt%	MeOH	3.2 (61 °C water)	130	11	6.7	0.078 GPa; 1.7%; 5.1 GPa	10
	RSF/water, 20–30% w/v	MeOH	No	Very weak	1.5	U.C.	very weak	11
	RSF/95% formic acid, 13% w/v	MeOH	3 (MeOH)	1077.3±173	29.3±11.9	39.9±6.1	0.295 GPa, 2.54%, 30.4 GPa	12
	RSF/TFA, 13% w/v	MeOH	3 (MeOH)	959.0±149.1	18.1±6.8	43.2	0.275 GPa, 1.33%, 28.1 GPa	12
	RSF/98% formic acid, 19% w/v	MeOH	5 (70 °C water)	257.5	16.4	5.5	very weak	13
	RSF/90% formic acid + 10% LiCl, 15 wt%	MeOH	4 (MeOH)	180	10	6.9	0.045 GPa, 1.2%, 4.0 GPa	14
	RSF//98% formic acid, 15% w/v	MeOH	4.5 (MeOH)	285.1±10.7	14.0±1.7	7.2	U.C.	15
	RSF/$CaCl_2$/formic acid, 12 wt%	Water	4	470.4±53.5	38.6±6.3	6.9±2.1	U.C.	16
	RSF/HFIP, 15 wt%	MeOH	4 (MeOH)	550	8.9	13.2	U.C.	17
	RSF/HFIP, 10 wt%	MeOH	3 (water) + steam annealing	193	19	5.2	U.C.	18
	RSF/HFA•3H_2O, 10 wt%	MeOH	3 (water) + steam annealing	321.2	16.1	5.3	U.C.	19
	RSF/NMMO-H_2O, 17 wt%	MeOH	7.2 (water)	320±20[d]	6±1[d]	12.2±0.3[d]	U.C.	20
	RSF/NMMO-H_2O, 17 wt%	MeOH	5.2 (water)	351±9[d]	7±1[d]	14.2±0.3[d]	0.046 GPa, 0.75%, 6.6 GPa[d]	21
	RSF/EMIMCl, 10 wt%	MeOH	2 (MeOH)	Brittle	U.C.	U.C.	U.C.	22
	RSF/NMMO•H_2O, 20 wt%	MeOH	3.6 (MeOH)	400	U.C.	U.C.	U.C.	23
	RSF/HFIP, 16% w/v	MeOH	3 (MeOH)	422±36	15±1.9	9.3±0.5	U.C.	24
	RSF/HFA, 16% w/v	MeOH	3 (MeOH)	295±36	18±3.7	9.8±1.1	U.C.	24
	RSF/HFIP, 12% w/v	MeOH	3 (MeOH)	400.5	20.7	4.3		25
	RSF/HFIP, 10 wt%	EtOH/ MeOH	U.C.	109.7	25	U.C.	U.C.	26
	RSF/NMMO•H_2O, 13 wt%	EtOH	2.7 (EtOH)	120	35	7.2	0.043 GPa, 2.2%, 2.6 GPa	27
	RSF/NMMO•H_2O, 17 wt%	EtOH	2.8 (EtOH)	127±8	12.7±1.9	5.3±0.2	U.C.	28
	RSF/NMMO•H_2O, U.C.	EtOH	2.0 (EtOH)	120±10	8.6±1.2	7.2±0.1	0.043 GPa, 1.1%, 3.8 GPa	29
	RSF/NMMO•H_2O, U.C.	EtOH	3.8 (EtOH)	127±8	12±2	5.3±0.2	0.043 GPa, 1.1%, 3.8 GPa	29
	RSF/water, 30% w/v	MeOH/CH_3COOH	3 (MeOH/CH_3COOH)	~400	~35	U.C.	~0.06 GPa, ~4%, U.C.	30
	RSF/ $CaCl_2$•H_2O, 8 wt%	PEG 30%	Wet-stretched	101±8	17±3	3±2	0.086 GPa, 124%, 1.0 GPa	31
	RSF/$CaCl_2$•H_2O, 16 wt%	EtOH:CH_3COOH 1 M (80:20)	2.4 and wet-stretched	250±20	30±10	9±4	0.052 GPa, 1.3%, 4.1 GPa	31
	RSF/ $CaCl_2$•H_2O, 16 wt%	isopropanol:CH_3COOH 1 M (80:20)	3.5 and wet-stretched	330±20	22±3	11±3	U.C.	31
Bombyx mori RSF dry spinning	RSF/water, 39 wt%	air	No	0.13	9.6	7.18	Very brittle	32
	RSF/$CaCl_2$/Water, 38-47wt%	air	4 (80 v/v% EtOH/water)	541.3±26.1	19.3±4.8	9.4±1.2	U.C.	33
	SF/$CaCl_2$/formic acid, 20 and 25% w/v	air	2 (EtOH)	333	35.1	8.8	0.123 GPa, 12.3%, U.C.	34
	RSF/ $CaCl_2$/water, 50wt%	microfluidic chip	2 (80 v/v% EtOH/water)	614	27	19	very brittle	35
	RSF/ $CaCl_2$/water, U.C.	air	2.0 (90% MeOH)	162.8±13.7	14.6±12.3	5.3±0.8	0.030 GPa, 3.6%, 1.2 GPa	36
	RSF/ $CaCl_2$/water, U.C.	air	2.0 (80% EtOH)	199.2±51.9	55.4±21.3	6.8±1.2	0.030 GPa, 3.6%, 1.2 GPa	36
	RSF/ $CaCl_2$/water, U.C.	air	2.0 (90% isopropanol aqueous solution)	188.5±35.9	4.4±1.0	4.8±1.8	0.030 GPa, 3.6%, 1.2 GPa	36
	RSF/ $CaCl_2$/water, U.C.	air	2.0 (Saturated $(NH_4)_2SO_4$ solution)	51.1±23.0	2.6±0.8	3.0±0.3	0.030 GPa, 3.6%,1.2 GPa	36
	RSF/ $CaCl_2$/water, 20wt%	air	1 (80 vol% EtOH)	63.9±13.5	7.5±2.3	1.7±0.7	0.003 GPa, 3.6%, 1.2 GPa	37

	RSF/ $CaCl_2$/water, 20wt%	air	2 (80 vol% EtOH)	199.8±143.4	55.4±21.3	6.4±6.1	0.003 GPa, 3.6%, 1.2 GPa	37
	RSF/ $CaCl_2$/water, 20wt%	air	3 (80 vol% EtOH)	301.5±70.6	35.8±21.9	6.2±1.7	0.003 GPa, 3.6%, 1.2 GPa	37
	RSF/ $CaCl_2$/water, 20wt%	air	EtOH	359	~55	U.C.	0.063 GPa, ~5%, U.C.	38
	RSF/$CaCl_2$/water, 40-60wt%	air	4 (80 vol% EtOH)	357.3±84.3	34.1±8.1	8.8	0.079 GPa, 10.6%, U.C.	39
	RSF/$CaCl_2$/water, 50wt%	air	4 (80 vol% EtOH)	337.7	24.6	11.1	0.046 GPa, U.C., U.C.	40
Regenerated Spider silk and recombinant protein spinning	Regenerated *N. edulis* spidroin/water, 0.08 wt%	air	No	110–140	10–27	6.0	All data are as-spun fibers	41
	N. clavipes spidroin/HFIP, 2.5 wt%	Acetone	3.5 (acetone)	320	4–8	8.0	very weak	42
	N. clavipes spodroin/HFIP, 289.4 kDa, 20% w/v	90% MeOH	5 (U. C.)	508±108	15±5	21±4	U.C.	43
	N. clavipes spidroin 1 (DP-1)/HFIP, U.C., 20%	isopropanol	2 (150 °C isopropanol)	140	103	4.6	U.C.	44
	A. diadematus ADF-3/water, 60kDa, 10-28% w/v	MeOH–water	5	269.6	43.4	13.2	U.C.	45
	N. clavipes Flag, MaSp like fusion proteins/HFIP, 58kDa, 25-30% w/v	90% isopropyl alcohol	U.C.	49.6±19.4	15.8±6.1	1.1±1.0	U.C.	46
	N. clavipes MaSp1/HFIP, 46kDa, 30% w/v	100% isopropanol	3 (75% isopropanol/water)	53.9±9.5	4.5±4.1	3.1±1.4	0.016 GPa, 1.46%, 1.06 GPa	47
	N. clavipes MaSp1/HFIP, 70kDa, 30% w/v	100% isopropanol	3 (75% isopropanol/water)	132.5±49.2	22.8±19.1	5.7±2.4	0.035 GPa, 3.13%, 2.78 GPa	47
	N. clavipes MaSp1MaSp2 4:1/HFIP, 30% w/v	isopropanol	3 (75% isopropanol/water)	37.6±20.3	53.9±68.0	3.4±1.1	0.023 GPa, 1.2%, 2.2 GPa	48
	N. clavipes MaSp1MaSp2 1:1/HFIP, 30% w/v	isopropanol	3 (75% isopropanol/water)	59.6±19.2	4.8±8.6	4.3±0.9	0.013GPa, 0.9%, 1.6 Gpa	48
	A. aurantia MaSp2/HFIP, 63kDa,10-12wt%	isopropanol	No	6.6±5.1	1.5±0.3	0.005	All data are as-spun fibers	49
	A. aurantia MaSp2/HFIP, 67kDa,10-12wt%	isopropanol	No	1.93±2.4	19±2.2	0.00004	All data are as-spun fibers	49
	A. aurantia MaSp2/HFIP, 71kDa, 10-12wt%	isopropanol	No	49.5±7.8	3.6±2.6	0.4±0.3	All data are as-spun fibers	49
	A. diadematus eADF3/water, 60kDa, 10–15% w/v	Water/isopropanol	6 (water/isopropanol)	370±59	110±25	4±1	0.054 GPa, 7%, 2 GPa	50
	N. clavipes MaSp1, MaSp2 inspired protein/HFIP, 50kDa, U. C.	isopropanol	5 (heat treatment in steam)	350±10[a]	42±2 [a]	6.3±0.2 [a]	U.C.	51
	N. clavipes MaSp2, Flag/HFIP, 58 kDa, 26-27% w/v	90% isopropyl alcohol/ 10% water coagulation bath	2-2.5 (90% v/v isopropyl alcohol)	127.5±23.0	52.3±23.6	4.4±1.0	U.C.	52
	N. clavipes MaSp2, Flag/HFIP, 62 kDa, 26-27% w/v	90% isopropyl alcohol/ 10% water coagulation bath	2-2.5 (90% v/v isopropyl alcohol)	96.2±28.8	29.6±20.5	3.8±2.1	0.023 GPa, 46.9%, 0.5 GPa	52
	Spider TuSp1/HFIP,45 kDa, 20% w/v	95% isopropanol	6 (75% isopropanol/water)	121.9±5	18±1	3.9	U.C.	53
	Spider TuSp1/HFIP,45 kDa, 20% w/v	95% isopropanol	3.5 (75% isopropanol/water)	95.1±3.3	25±4	2.6	U.C.	53
	N. clavipes flagelliform silk-like protein/HFIP, 66 kDa, 15% w/v	isopropanol	3 (80% isopropanol/water)	150.6±31.3	84.5±37.8	4	0.026 GPa, 0.66%, U.C.	54
	Engineered large spider eggcase silk/HFIP, 378 kDa, 8-10 wt%	$ZnCl_2$ and $FeCl_3$ in water	5 (50-70% ethanol)	308±57	9.6±3	9.3±3	Breaking strength smaller than 0.1 GPa	55
	A. aurantia MaSp 2/HFIP, 80 kDa, 56% w/v	isopropanol	4-6 (85% isopropanol)	27.1±12.5	22.0±26	1.52±0.49	0.010 GPa, 1.5%, 0.91 GPa	56
	A. aurantia MaSp 2/HFIP, 80 kDa, 56% w/v	isopropanol	4 (85% isopropanol at 60 °C)	29.0±14.1	27.3±14.0	1.51±0.59	0.010 GPa, 1.5%, 0.91 GPa	56
	A. aurantia MaSp 2/HFIP, 91 kDa, 45-60% w/v	isopropanol	2 (85% isopropanol)	14.6±6.3	40.2±40.4	1.42±0.41	0.006 GPa, 1.9%, 0.47 GPa	56
	A. aurantia MaSp 2/HFIP, 91 kDa, 45-60% w/v	isopropanol	3-4 (85% isopropanol at 60 °C)	14.0±7.4	71.6±82.0	0.98±0.54	0.006 GPa, 1.9%, 0.47 GPa	56

	A. aurantia MaSp 2/HFIP, 86.5 kDa, 45-60% w/v	isopropanol	3 (75% isopropanol)	27.9±11.9	93.0±67.1	1.79±0.59	0.013 GPa, 1.2%, 1.88 GPa	56
	A. aurantia MaSp 2/HFIP, 86.5 kDa, 45-60% w/v	isopropanol	4 (75% isopropanol at 60 °C)	39.0±7.4	181.3±103.5	1.60±0.35	0.013 GPa, 1.2%, 1.88 GPa	56
	A. aurantia MaSp 2/HFIP, 86.5 kDa, 45-60% w/v	isopropanol	3 (75% EtOH)	46.9±16.8	3.8±4.1	3.62±1.53	0.013 GPa, 1.2%, 1.88 GPa	56
	A. aurantia MaSp 2/HFIP, 86.5 kDa, 45-60% w/v	isopropanol	4 (85% EtOH at 60 °C)	53.5±18.0	18.0±21.6	2.90±1.10	0.013 GPa, 1.2%, 1.88 GPa	56
	*N. clavipes*MaSp1 and MaSp2 analog/(HFIP+>88% formic acid in 4:1 ratio, 65 kDa, 25% w/v	isopropanol	1.5/2 (10:90 isopropanol:water)	221.7±11	56±6.6	U.C.	0.032 GPa, 1.1%, U.C.	57
	Recombinant MaSp1 and MaSp 2/water, 50-75 kDa, 12% w/v	isopropanol	2-2.5 (80% isopropanol)	192.2±51.5	28.1±26	8.3	U.C.	58
	N. clavipes MaSp 1/(NaCl/water), 47 kDa, 12% w/v	ethanol	U.C.	62.3±17.2	3.5±1.2	4±2.8	U.C.	59
	N. clavipes MaSp 1/(NaCl/water), 47 kDa, 10-17% w/v	ethanol	U.C.	286.2±137.7	18.3±12.8	8.4±4.3	U.C.	59
	Euprosthenops australis MiSp 1/(aqueous buffer at pH 8), 33 kDa, 50% w/v	Aqueous solution (sodium acetate, pH 2.5-7.5)	0	162±8	37±5	6±0.8	All data are as-spun fibers	60

[a] The blue and red regions are mechanical properties of regenerated silkworm silk fibers, the blue and red region are spun by wet-spinning and dry-spinning method, respectively. The green region is mechanical properties of regenerated and recombinant spider silk fibers. Some data are direct extract from reference (61 and 62). [b] Spinning dope is expressed as "solute/solvent, concentration", [c] The mechanical properties of as-spun fibers (without any post-treatments) are expressed as: stress (GPa), strain (%), modulus (GPa). [d] these values are calculated from true stress/strain. RSF = regenerated silk fibroin; ADF-3= *Araneus diadematus* (MaSp2) fibroin 3; eADF-3= engineered variants of *Araneus diadematus* (MaSp2) fibroin 3; MaSp=Major ampullate spidroin; MiSp=Minor ampullate spidroin; TuSp=Tubuliform spidroin; TFA= Trifluoroacetic acid; HFIP= Hexafluoroisopropanol; NMMO= N-methylmorpholine-N-oxide; HFA= Hexafluoroacetone trihydrate; EMIMCl= 1-Ethyl-3-methylimidazolium chloride; U.C. = unclear.

Supplementary Table 2. Structures and mechanical properties of as-spun SNFs.

Category NO.	Reeling speed mm s^{-1}	Average cross-sectional area (mm^2)[a]	β-sheet content (%)[b]	Stress	Strain (%)	Modulus (GPa)	Toughness (MJ m^{-3})
1	14	0.002±0.001	45±3	93±31	4.5±3.8	11±4	2.4±2.4
2	10	0.007±0.001	43±1	98±45	9.4±7.6	10±5	6.8±4.9
3	8	0.014±0.003	38±2	133±35	8.1±5.3	11±3	8.8±5.0
4	6	0.018±0.002	36±2	106±44	8.1±7.2	9±3	8.2±9.5
5	4	0.024±0.003	34±5	109±34	14.0±4.9	8±1	13.9±9.2

[a] The average cross-sectional areas were calculated from SEM images. [b] β-sheet contents were calculated from deconvolution of amide I band of RSFs

Supplementary references:

1. Zhou H., Shao Z., Chen X. Wet-spinning of regenerated silk fiber from aqueous silk fibroin solutions: Influence of calcium ion addition in spinning dope on the performance of regenerated silk fiber. *Chin. J. Polym. Sci.* **32**, 29-34 (2014).

2. Zhou G, Shao Z, Knight D. P., Yan J., Chen X. Silk fibers extruded artificially from aqueous solutions of regenerated *Bombyx mori* silk fibroin are tougher than their natural counterparts. *Adv. Mater.* **21**, 366-370 (2009).

3. Yan J., Zhou G., Knight D. P., Shao Z., Chen X. Wet-spinning of regenerated silk fiber from aqueous silk fibroin solution: discussion of spinning parameters. *Biomacromolecules* **11**, 1-5 (2010).

4. Yazawa S. Spinning of concentrated aqueous silk fibroin solution. *J. Chem. Soc. Jpn.* **63**, 1428−1430 (1960).

5. Ishizaka, H., Watanabe, Y., Ishida, K., Fukumoto, O. Regenerated silk prepared from ortho phosphoric acid solution of fibroin. *J. Seric. Sci. Jpn*. **58**, 87–95 (1989).

6. Ling S., Zhou L., Zhou W., Shao Z., Chen X. Conformation transition kinetics and spinnability of regenerated silk fibroin with glycol, glycerol and polyethylene glycol. *Mater. Lett.* **81**, 13-15 (2012).

7. Fang G, *et al*. Insights into silk formation process: correlation of mechanical properties and structural evolution during artificial spinning of silk fibers. *ACS Biomater. Sci. Eng*. **2**, 1992-2000 (2016).

8. Chen Z., Zhang H., Lin Z., Lin Y., van Esch J. H., Liu X. Programing performance of silk fibroin materials by controlled nucleation. *Adv. Funct. Mater*. **26**, 8978-8990 (2016).

9. Sohn S., Gido S. P. Wet-spinning of osmotically stressed silk fibroin. *Biomacromolecules* **10**, 2086-2091 (2009).

10. Matsumoto K., Uejima H., Iwasaki T., Sano Y., Sumino H. Studies on regenerated protein fibers .3. Production of regenerated silk fibroin fiber by the self-dialyzing wet spinning method. *J. Appl. Polym. Sci.* **60**, 503-511 (1996).

11. Ha S. W., Park Y. H., Hudson S. M. Dissolution of *Bombyx mori* silk fibroin in the calcium nitrate tetrahydrate-methanol system and aspects of wet spinning of fibroin solution. *Biomacromolecules* **4**, 488-496 (2003).

12. Ha S. W., Gracz H. S., Tonelli A. E., Hudson S. M. Structural study of irregular amino acid sequences in the heavy chain of Bombyx mori silk fibroin. *Biomacromolecules* **6**, 2563-2569 (2005).

13. Um I. C., Ki C. S., Kweon H. Y., Lee K. G., Ihm D. W., Park Y. H. Wet spinning of silk polymer - II. Effect of drawing on the structural characteristics and properties of filament. *Int J. Biol. Macromol.* **34**, 107-119 (2004).

14. Lock R. L. Process for spinning polypeptide fibers. *US Pat.* 5171505 (1992).

15. Ki C. S., Kim J. W., Oh H. J., Lee K. H., Park Y. H.. The effect of residual silk sericin on the structure and mechanical property of regenerated silk filament. *Int. J. Biol. Macromol.* **41**, 346-353 (2007).

16. Zhang F., *et al*. Regeneration of high-quality silk fibroin fiber by wet spinning from $CaCl_2$–formic acid solvent. *Acta Biomater.* **12**, 139-145 (2015).

17. Lock R. L. Process for making silk fibroin fibers. *US Pat.* 5252285 (1993).

18. Zhao C. H., Yao J. M., Masuda H., Kishore R., Asakura T. Structural characterization and artificial fiber formation of Bombyx mori silk fibroin in hexafluoro-iso-propanol solvent system. *Biopolymers* **69**, 253-259 (2003).

19. Yao J. M., Masuda H., Zhao C. H., Asakura T. Artificial spinning and characterization of silk fiber from Bombyx mori silk fibroin in hexafluoroacetone hydrate. *Macromolecules* **35**, 6-9 (2002).

20. Plaza G. R., *et al.* Old silks endowed with new properties. *Macromolecules* **42**, 8977-8982 (2009).

21. Plaza G. R., *et al.* Correlation between processing conditions, microstructure and mechanical behavior in regenerated silkworm silk fibers. *J. Polym. Sci. Part B Polym. Phys.* **50**, 455-465 (2012).

22. Phillips D. M., *et al.* Regenerated silk fiber wet spinning from an ionic liquid solution. *J. Mater. Chem.* **15**, 4206-4208 (2005).

23. Xu Y., Shao H. L., Zhang Y. P., Hu X. C. Studies on spinning and rheological behaviors of regenerated silk fibroin/N-methylmorpholine-N-oxide center dot H2O solutions. *J. Mater. Sci.* **40**, 5355-5358 (2005).

24. Zhu Z., *et al.* Mechanical properties of regenerated *Bombyx mori* silk fibers and recombinant silk fibers produced by transgenic silkworms. *J. Biomater. Sci., Polym. Ed.* **21**, 395-411 (2010).

25. Zhu Z., Ohgo K., Watanabe R., Takezawa T., Asakura T. Preparation and characterization of regenerated Bombyx mori silk fibroin fiber containing recombinant cell-adhesive proteins; nonwoven fiber and monofilament. *J. Appl. Polym. Sci.* **109**, 2956-2963 (2008).

26. Zuo B., Liu L., Wu Z. Effect on properties of regenerated silk fibroin fiber coagulated with aqueous methanol/ethanol. *J. Appl. Polym. Sci.* **106**, 53-59 (2007).

27. Marsano E., Corsini P., Arosio C., Boschi A., Mormino M., Freddi G. Wet spinning of Bombyx mori silk fibroin dissolved in N-methyl morpholine N-oxide and properties of regenerated fibres. *Int. J. Biol. Macromol.* **37**, 179-188 (2005).

28. Corsini P., *et al.* Influence of the draw ratio on the tensile and fracture behavior of NMMO regenerated silk fibers. *J. Polym. Sci. Pol. Phys.* **45**, 2568-2579 (2007).

29. Plaza G. R., Corsini P., Perez-Rigueiro J., Marsano E., Guinea G. V., Elices M. Effect of water on Bombyx mori regenerated silk fibers and its application in modifying their mechanical properties. *J. Appl. Polym. Sci.* **109**, 1793-1801 (2008).

30. Zhu Z., Imada T., Asakura T. Preparation and characterization of regenerated fiber from the aqueous solution of Bombyx mori cocoon silk fibroin. *Mater. Chem. Phys.* **117**, 430-433 (2009).

31. Madurga R., Gañán-Calvo A. M., Plaza G. R., Guinea G. V., Elices M., Pérez-Rigueiro J. Production of high performance bioinspired silk fibers by straining flow spinning. *Biomacromolecules* DOI: 10.1021/acs.biomac.6b01757 (2017).

32. Xie F, Zhang HH, Shao HL, Hu XC. Effect of shearing on formation of silk fibers from regenerated Bombyx mori silk fibroin aqueous solution. *Int. J. Biol. Macromol.* **38**, 284-288 (2006).

33. Peng Q., Shao H., Hu X., Zhang Y. Role of humidity on the structures and properties of regenerated silk fibers. *Prog. Nat. Sci. Mater. Int.* **25**, 430-436 (2015).

34. Yue X., Zhang F., Wu H., Ming J., Fan Z., Zuo B. A novel route to prepare dry-spun silk fibers from CaCl2–formic acid solution. *Mater. Lett.* **128**, 175-178 (2014).

35. Luo J., *et al.* Tough silk fibers prepared in air using a biomimetic microfluidic chip. *Int. J. Biol. Macromol.* **66**, 319-324 (2014).

36. Wei W., Zhang Y., Zhao Y., Shao H., Hu X. Studies on the post-treatment of the dry-spun fibers from regenerated silk fibroin solution: Post-treatment agent and method. *Mater. Des.* **36**, 816-822 (2012).

37. Wei W., Zhang Y., Shao H., Hu X. Posttreatment of the dry-spun fibers obtained from regenerated silk fibroin aqueous solution in ethanol aqueous solution. *J. Mater. Res.* **26**, 1100 (2011).

38. Wei W., Zhang Y., Zhao Y., Luo J., Shao H., Hu X. Bio-inspired capillary dry spinning of regenerated silk fibroin aqueous solution. *Mater. Sci. Eng. C* **31**, 1602-1608 (2011).

39. Jin Y., Zhang Y., Hang Y., Shao H., Hu X. A simple process for dry spinning of regenerated silk fibroin aqueous solution. *J. Mater. Res.* **28**, 2897-2902 (2013).

40. Sun M., Zhang Y., Zhao Y., Shao H., Hu X. The structure-property relationships of artificial silk fabricated by dry-spinning process. *J. Mater. Chem.* **22**, 18372-18379 (2012).

41. Shao Z., Vollrath F., Yang Y., Thogersen H. C. Structure and behavior of regenerated spider silk. *Macromolecules* **36**, 1157-1161 (2003).

42. Seidel A., *et al.* Regenerated spider silk: Processing, properties, and structure. *Macromolecules* **33**, 775-780 (2000).

43. Xia X., Qian Z., Ki C. S., Park Y. H., Kaplan D. L., Lee S. Y. Native-sized recombinant spider silk protein produced in metabolically engineered Escherichia coli results in a strong fiber. *Proc. Natl. Acad. Sci. U.S.A.* **107**, 14059-14063 (2010).

44. Fahnestock S. R. Recombinantly produced spider silk. *US Pat.* 6268169 (2001).

45. Lazaris A., *et al.* Spider silk fibers spun from soluble recombinant silk produced in mammalian cells. *Science* **295**, 472-476 (2002).

46. Teulé F., Furin W. A., Cooper A. R., Duncan J. R., Lewis R. V. Modifications of spider silk sequences in an attempt to control the mechanical properties of the synthetic fibers. *J. Mater. Sci.* **42**, 8974-8985 (2007).

47. An B., Hinman M. B., Holland G. P., Yarger J. L., Lewis R. V. Inducing β-Sheets formation in synthetic spider silk fibers by aqueous post-spin stretching. *Biomacromolecules* **12**, 2375-2381 (2011).

48. An B., *et al.* Reproducing natural spider silks' copolymer behavior in synthetic silk mimics. *Biomacromolecules* **13**, 3938-3948 (2012).

49. Brooks A. E., Stricker S. M., Joshi S. B., Kamerzell T. J., Middaugh C. R., Lewis R. V. Properties of synthetic spider silk fibers based on *Argiope aurantia* MaSp2. *Biomacromolecules* **9**, 1506-1510 (2008).

50. Heidebrecht A., *et al.* Biomimetic fibers made of recombinant spidroins with the same toughness as natural spider silk. *Adv. Mater.* **27**, 2189-2194 (2015).

51. Elices M., *et al.* Bioinspired fibers follow the track of natural spider silk. *Macromolecules* **44**, 1166-1176 (2011).

52. Teulé F., *et al.* Combining flagelliform and dragline spider silk motifs to produce tunable synthetic biopolymer fibers. *Biopolymers* **97**, 418-431 (2012).

53. Gnesa E., *et al.* Conserved C-terminal domain of spider tubuliform spidroin 1 contributes to extensibility in synthetic fibers. *Biomacromolecules* **13**, 304-312 (2012).

54. Adrianos S. L., *et al. Nephila clavipes* flagelliform silk-like GGX motifs contribute to extensibility and spacer motifs contribute to strength in synthetic spider silk fibers. *Biomacromolecules* **14**, 1751-1760 (2013).

55. Lin Z., Deng Q., Liu X. -Y., Yang D. Engineered large spider eggcase silk protein for strong artificial fibers. *Adv. Mater.* **25**, 1216-1220 (2013).

56. Albertson A. E., Teulé F., Weber W., Yarger J. L., Lewis R. V. Effects of different post-spin stretching conditions on the mechanical properties of synthetic spider silk fibers. *J. Mech. Behav. Biomed. Mater.* **29**, 225-234 (2014).

57. Copeland C. G., Bell B. E., Christensen C. D., Lewis R. V. Development of a process for the spinning of synthetic spider silk. *ACS Biomater. Sci. Eng.* **1**, 577-584 (2015).

58. Jones J. A., *et al.* More than just fibers: An aqueous method for the production of innovative recombinant spider silk protein materials. *Biomacromolecules* **16**, 1418-1425 (2015).

59. Peng Q., *et al.* Recombinant spider silk from aqueous solutions via a bio-inspired microfluidic chip. *Sci. Rep.* **6**, 36473 (2016).

60. Andersson M., *et al.* Biomimetic spinning of artificial spider silk from a chimeric minispidroin. *Nat. Chem. Biol.* **13**, 262-264 (2017).

61. Koeppel A., Holland C. Progress and trends in artificial silk spinning: A systematic review. *ACS Biomater. Sci. Eng.* **3**, 226-237 (2017).

62. Fu C. J., Shao Z., Fritz V. Animal silks: their structures, properties and artificial production. *Chem. Commun.* **43**, 6515-6529 (2009).
